# Risk factors associated with the infection of sheep with *Dichelobacter nodosus*

**DOI:** 10.1038/s41598-022-13933-4

**Published:** 2022-06-15

**Authors:** Julia Storms, Anna Wirth, Danae Vasiliadis, Jörg Jores, Peter Kuhnert, Ottmar Distl

**Affiliations:** 1 Institute of Animal Breeding and Genetics, University of Veterinary Medicine Hannover, 30559 Hannover, Germany; 2 Institute of Veterinary Bacteriology, Vetsuisse Faculty, University of Bern, 3012 Bern, Switzerland

**Keywords:** Diseases, Medical research, Risk factors, Signs and symptoms

## Abstract

Ovine footrot is a highly contagious foot disease caused by the gram-negative bacterium *Dichelobacter nodosus *(*D. nodosus*). In a recent report, we showed a prevalence of 42.9% *D. nodosus* positive swabs across Germany. In this follow-up study, we used real-time PCR results for *D. nodosus* and footrot scores of 9297 sheep from 208 flocks and collated these data with survey data on herd and animal characteristics and herd management. The aims of the present study were to investigate herd and animal factors associated with *D. nodosus* infection and footrot scores in individual sheep. Multivariable analyses with generalized mixed models showed that month of recording, breed, herdbook membership, use of antibiotics, and footbaths in the past 3–10 years, signs of footrot in the past 12 months and flock environment of the sheep, modelled as a random farm effect within region, were significant risk factors. Among the 21 different breeds, Romney had the lowest risk of *D. nodosus* infection, while Swifter had the highest risk and German Merino and German White Heath were the next breeds at highest risk of *D. nodosus* infection. The variance between farms in the prevalence of *D. nodosus* was large and accounted for 84% of the total variance in the mixed model analysis. We conclude that specific and as yet unknown effects influencing *D. nodosus* infections in flocks, as well as breed and weather, are the most important effects on *D. nodosus* infection in sheep, pointing towards the need to establish adequate infection control at farm level.

## Introduction

The gram-negative, anaerobic and aerotolerant bacterium *Dichelobacter nodosus *(*D. nodosus*) is the aetiological agent of ovine footrot^1,2^. Footrot is a highly contagious disease of sheep, which are the main host, causing lameness associated with severe pain^3^ and production losses. To a lesser extent other ruminants like goats, cattle, New World camelids, and wild animals such as ibex and mouflon can be affected or carry *D. nodosus*^4,5^. The bacterium mainly spreads from infected to non-infected animals through contaminated pasture^6^. Furthermore, cross-infection between sheep and co-grazing cattle has been reported^4^. However, the main route of infection is from sheep to sheep either through contact between flocks, the environment or vectors^7–9^.

The broad spectrum of footrot lesions is associated with different clinical manifestations of *D. nodosus* infection. Mild cases and the onset of severe cases are manifested as an interdigital dermatitis with loss of hair and inflamed skin^10,11^. Later stages are characterized by a separation of the hoof horn from the underlying tissue, starting at the axial wall of the hoof and being accompanied by a foul smell^12^. The underrunning of the hoof horn can extend to the abaxial wall and the toe of the hoof, consequently resulting in the total loss of the hoof capsule^13^ The severity of clinical signs is determined by three main factors: the pathogens’ virulence, the environmental conditions and the individual genetic predisposition of the host^12^. Virulent strains of *D. nodosus* can be distinguished from benign ones based on the produced extracellular proteases (AprV2 and AprB2, respectively). A 2-bp substitution in the corresponding *aprV2* and *aprB2* genes discriminates the two virulotypes of *D. nodosus*^14^. Thus, a highly sensitive and specific competitive real-time PCR to detect *D. nodosus* and to discriminate the virulotypes was developed by Stäuble et al.^15^ and successfully applied in many studies^16–19^. A warm and moist environment provides optimal conditions for *D. nodosus* to multiply and spread^20^. Furthermore, pre-existing microlesions of the interdigital skin due to water-maceration, sharp grass or similar causes foster invasion of the dermis with *D. nodosus*^1,21^. The innate genetic makeup of the animal, i.e. the breed, influences the immune response to the infection with pathogens such as *D. nodosus,* and has therefore been the objective of several studies. Likewise, it is possible that the skin barrier has an effect on infection due to a certain resistance to microtraumas. Emery et al. demonstrated that British breeds are more resistant to footrot than Merinos^22^. Especially Romney sheep showed a superior immune response compared to Merinos^23^. Seven on chromosome-level significant SNPs were detected in a genome-wide association study for footrot in Texel sheep^24^. The aim of New Zealand’s breeding programme is the development of genomic breeding values for footrot resistance in fine-wool sheep through extensive genotyping using ovine 50 K SNP chips^25^.

Recently, we demonstrated a high prevalence of *D. nodosus* across a large number of German sheep flocks, with an overall prevalence of 71.4% and 42.9% at flock and animal level, respectively. Virulent *D. nodosus* was detected in more than 90% of the *D. nodosus*-positive swabs, while benign strains were detected in only 4.91%. Only virulent and only benign *D. nodosus* strains were found in 55.5% and 3.4% of the flocks, respectively. Furthermore, the within-flock prevalence of *D. nodosus* varied significantly from 0 to 100%^26^. The aim of this follow-up study was to evaluate risk factors for animals associated with *D. nodosus* infection based on real-time PCR results and footrot scores. During each farm visit, data were collected on farm and animal variables, farm management, observations of footrot in the last 12 months and the last 3–10 years, and the type of treatment given to the affected sheep. These data should allow evaluation of associations between farm and animal variables on the one hand and *D. nodosus* infection and footrot scores on the other hand. Due to the large number of herds and animals across Germany, farm and animal variables were not confounded with each other, so that we could employ multivariable models to evaluate the effects of risk factors.

## Results

### Descriptive summary of dataset

#### Overall prevalence and region

In total, the results of 208 interviews with sheep farmers were included in the study and 9297 samples (interdigital 4-feet skin swabs) were tested for *D. nodosus* using a competitive real-time PCR, which can distinguish benign from virulent strains. We did not detect *D. nodosus* in 3586 out of 9297 samples (38.57%), while benign *D. nodosus* (*aprB2*+) was detected in 188 out of 9297 samples (2.02%). Virulent *D. nodosus* (*aprV2*+) were detected in 5328 samples (57.31%), and 195 samples (2.10%) were positive for both virulotypes (Table S1). The numbers, frequencies, and respective 95% confidence intervals (95%-CI) of sheep tested positive for *D. nodosus* (*aprV2*+ and/or *aprB2*+) and virulent *D. nodosus* (*aprV2*+) in the different levels of each source of variation are presented in Table 1. In Northern Germany, 42.21% of the sampled sheep tested positive for *D. nodosus* (both virulotypes) and 36.86% tested positive for virulent *D. nodosus*. In Southern Germany more than half of all sampled sheep (52.44%) tested positive for *D. nodosus* (both virulotypes).

**Table 1:** Number, frequencies (%) and 95% confidence intervals (CI) of sheep tested positive for *D. nodosus* (*aprV2*+ and/or *aprB2*+) and positive for virulent *D. nodosus* (*aprV2*+) by real-time PCR in the different levels of each source of variation.

Source of variation	Level	Number of sheep	*D. nodosus*-positive sheep (*aprV2*+ and/or *aprB2*+)			Virulent *D. nodosus*-positive sheep (*aprV2* +*)*		
			Frequency	CI		Frequency	CI	
Region	North	4238	42.21	40.72	43.72	36.86	35.40	38.33
	East	3463	38.78	37.15	40.43	34.33	32.75	35.94
	South	1596	52.44	49.96	54.92	52.32	49.83	54.79
Months	Jan–Feb	1204	49.83	46.97	52.70	47.76	44.90	50.62
	Mar–Apr	1858	32.62	30.49	34.80	26.10	24.12	28.16
	May–Jun	1898	50.21	47.94	52.48	49.68	47.41	51.96
	Jul–Aug	999	37.84	34.82	40.93	34.93	31.98	37.98
	Sep–Oct	1399	24.73	22.49	27.08	21.44	19.32	23.69
	Nov–Dec	1939	56.01	53.77	58.23	48.17	45.92	50.42
Herdbook	Yes	4885	31.34	30.04	32.66	24.01	22.82	25.24
	No	4412	55.26	53.78	56.73	54.69	53.21	56.17
Year	2019	3369	54.70	53.01	56.40	49.27	47.57	50.98
	2020	5928	35.86	34.64	37.10	32.49	31.30	33.70
Sex	Male	1375	27.71	25.36	30.16	25.02	22.75	27.40
	Female	7922	45.29	44.19	46.40	40.92	39.84	42.02
Flock size	< 41	762	30.62	28.12	33.20	29.15	26.69	31.71
	41–100	1724	44.95	42.68	47.24	41.85	39.60	44.12
	101–200	1922	31.58	28.89	34.37	30.09	27.44	32.84
	201–500	2525	31.04	28.80	33.35	19.85	17.94	21.88
	> 500	2364	55.52	53.82	57.21	52.34	50.63	54.04
Antibiotics (3–10)	Yes	495	35.96	31.73	40.36	27.27	23.39	31.43
	No	237	29.11	23.41	35.35	29.11	23.41	35.35
	No Response	8565	43.46	42.40	44.51	39.49	38.45	40.53
Footbaths (3–10)	Yes	276	32.97	27.45	38.86	27.54	22.35	33.21
	No	462	40.69	36.18	45.33	33.55	29.25	38.06
	No Response	8559	43.11	42.06	44.17	39.20	38.16	40.24
Footrot (12)	Yes	4535	59.40	57.96	60.84	57.95	56.50	59.39
	No	3346	23.43	22.00	24.90	18.47	17.17	19.83
	No Response	1416	34.68	32.19	37.22	24.01	21.81	26.32

#### Time: year and month of recording

**Figure 1:**
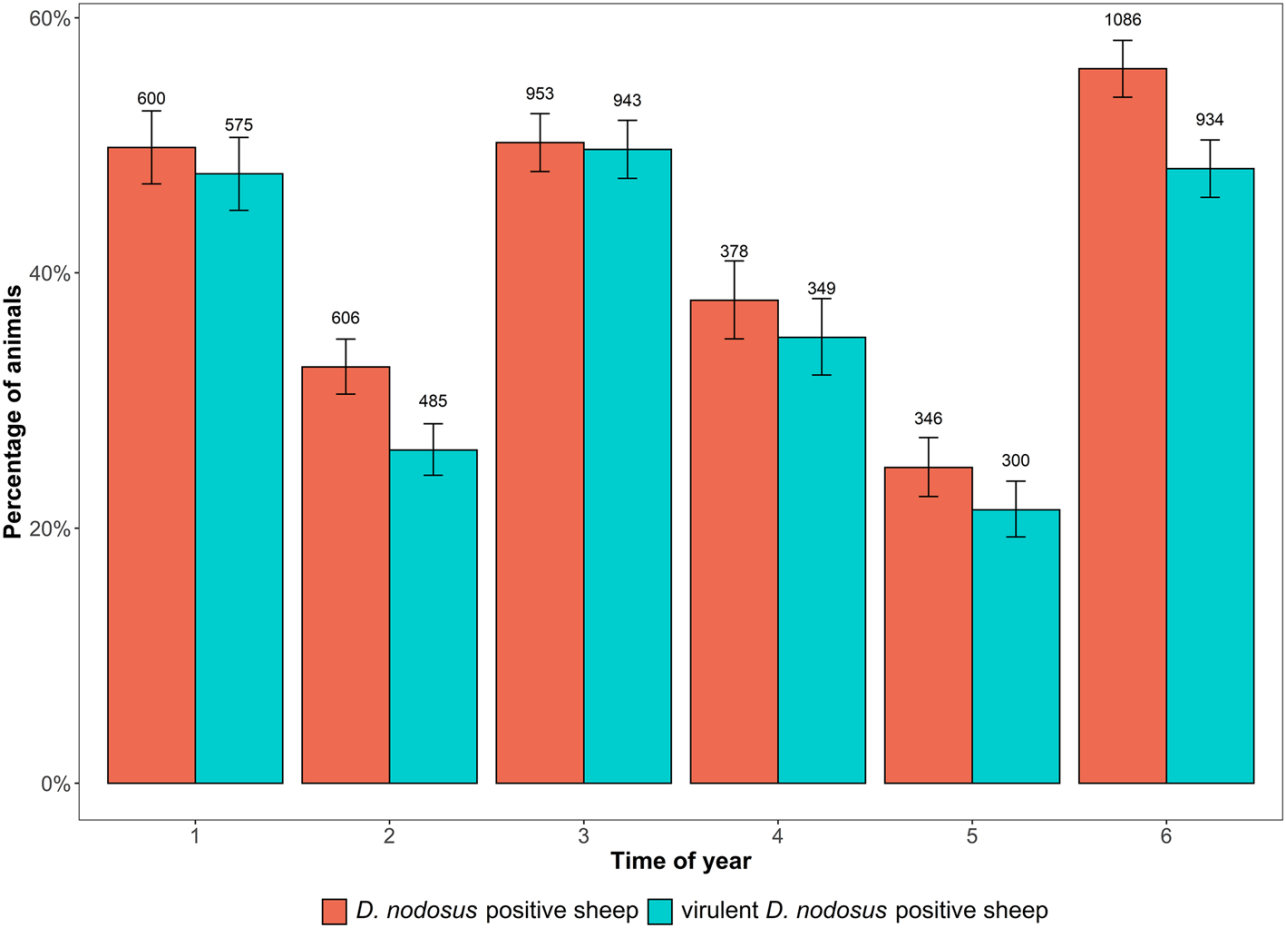
Percentage of animals tested positive for *D. nodosus* (*aprV2*+ and/or *aprB2*+) (red) and for virulent *D. nodosus* (*aprV2*+) (blue) at different times of the year, with 1 = Jan–Feb, 2 = Mar–Apr, 3 = May–Jun, 4 = Jul–Aug, 5 = Sep–Oct, 6 = Nov–Dec. Confidence intervals (95%-CI) and the total number of sampled sheep are also presented.

In 2020, more sheep were sampled (63.76% vs. 36.24%), but in 2019, more sheep were *D. nodosus*-positive (54.70% vs. 35.86%). Most *D. nodosus*-positive sheep were detected in November and December (56.01%), and the fewest in September and October (15.05%) (Fig. 1). The highest percentage of sheep showing only virulent *D. nodosus* was recorded in May and June (49.68%).

#### Herdbook, sex and flock size

In total, 52.54% of all sheep included in the study were kept on farms with herdbook affiliation, but only 24.01% of those animals tested positive for virulent *D. nodosus*, compared to 54.69% of sheep kept by non-herdbook breeders. Most of the sheep tested were female (85.21%), and they also showed a higher prevalence of *D. nodosus* (45.29%) and virulent *D. nodosus* (40.92%), compared to male sheep (27.71% and 25.02%, respectively). Most *D. nodosus*-positive sheep were found in large flocks with > 500 animals per flock (55.52%), while in small flocks with < 41 animals per flock only 30.62% of animals tested positive for *D. nodosus*. Most benign *D. nodosus* were detected in flocks with 201–500 animals per flock, a similar trend towards higher detection rates in large flocks as for the virulent strains.

#### Management factors: antibiotics and footbaths, clinical footrot in the past

More sheep were infected on farms that had used antibiotics to treat footrot in the 3–10 years prior to sampling than on farms that did not treat their animals with antibiotics (35.96% vs. 29.11%). Fewer *D. nodosus*-positive sheep were found if they had been treated with footbaths against footrot in the past 3–10 years (32.97% vs. 40.69%). *D. nodosus* and virulent *D. nodosus* were detected in 59.4% and 57.95% of samples, when the farmer reported that clinical signs of footrot had occurred in the flock within the last 12 months. If there were no clinical signs of footrot in the flock in the last 12 months, 23.43% and 18.47% of the samples tested positive for *D. nodosus* and virulent *D. nodosus*.

### Risk factor analysis

#### Univariable analyses

The results of the univariable analyses for the trait “infection with *D. nodosus”* (*aprV2*+ and/or *aprB2*+) showed a p-value < 0.05 for the sources of variation “month of recording”, “herdbook member”, “breed”, “treatment of diseased sheep with antibiotics in the previous 3–10 years”, “treatment of sheep with footbaths in the previous 3–10 years” and “clinical footrot on animals in the flock within the last 12 months” (Table S2). Other species like “goats”, “cattle”, “donkeys” and “horses” present on the farm had no significant effect. The factors “number of rams on the farm”, “flock size”, “sex of the animal” and “age of the animal” also showed p-values > 0.05. The analyses of risk factors for the trait “infection with virulent *D. nodosus”* (*aprV2*+) showed similar results. However, the factor “sex of the animal” had also a significant effect in this case (p = 0.0327) (Table S3). The univariable associations with the development of footrot scores are presented in Table S4. In the analysis for the categorical trait “footrot score” the following factors have a significant effect: “month of recording”, “herdbook member”, “year”, “sex”, “Horses on the farm”, “clinical signs of footrot on animals in the flock within the last 12 months” and “breed”.

#### Multivariable analyses

The results of the final model with the significant effects of the respective risk factors are presented in Table 2. All effects except “region” remained significant (p < 0.05) for the presence of *D. nodosus.* The effects “region” and “treatment of diseased sheep with antibiotics in the previous 3–10 years” showed p-values > 0.05 in the final model for the presence of virulent *D. nodosus.* Only the effects “month of recording”, “year”, “age of the animal”, “sex of the animal”, “clinical footrot within the last 12 months” and “breed” remained significant in the final model for footrot scores. The odds ratios (OR) and 95%-confidence intervals (95%-CI) for the footrot scores found on sheep at the time of sampling were analysed using an ordered multinomial distribution function and cumulative logits as link function (Table S5). For each risk factor in the multivariable final model one level was chosen as reference with an OR of 1. There is a higher risk for sheep to have higher footrot scores in the months March, April, July and August compared to January and February (OR > 7). Adult sheep have a higher risk for high footrot scores compared to lambs (OR > 1).

**Table 2:** Results of the final generalized mixed linear model using a binomial distribution function and logit as link function with the significant effects (p-value < 0.05), their degrees of freedom (DF) and F-values associated with the presence of *D. nodosus* (*aprV2*+ */aprB2* +) and only virulent *D. nodosus* (*aprV2*+) in the individual sheep.

Source of variation	DF	All *D. nodosus*		Only *aprV2* + *D. nodosus*		Footrot scores	
		F-value	P-value	F-value	P-value	F-value	P-value
Region	2	1.21	0.3008	2.76	0.0659	1.18	0.3100
Month	5	12.29	< 0.0001	4.36	0.0006	5.30	< 0.0001
Year	1	NI	NI	6.34	0.0118	37.39	< 0.0001
Herdbook member	1	5.36	0.0207	8.55	0.0035	NI	NI
Breed	21	6.69	< 0.0001	7.25	< 0.0001	9.00	< 0.0001
Sex	1	NI	NI	7.16	0.0075	26.80	< 0.0001
Age	1	NI	NI	NI	NI	12.08	0.0005
Antibiotics (3–10)	2	3.09	0.0456	3.00	0.0144	NI	NI
Footbaths (3–10)	2	6.30	0.0019	4.25	0.0144	NI	NI
Footrot (12)	2	3.81	0.0222	5.30	0.0050	3.00	0.0497

*NI* not included in the final model.

An ordered multinomial distribution function and cumulative logit as link function was used in the final generalized mixed linear model analysis of footrot scores.

#### Farm effect

The analysis of covariance parameters of farms within regions as random effect and other factors as fixed effects in the generalized mixed models, including standard errors (SE), Z- and p-values are presented in Table 3. It shows that 84% of the random variance between farms is due to the individuality of each farm, which has the most substantial impact on the infection status of an animal with *D. nodosus*. This individuality is made up of a combination of several factors that influence the prevalence of *D. nodosus* infections or the distribution of footrot scores in the herd and are not accounted for by other effects in the generalized linear mixed model, such as weather by farm soil type, pasture and specific farm management practices.

**Table 3:** Variance parameter estimates with their standard errors (SE), Z-values and p-values for the variables infection with *D. nodosus* (*aprV2*+ */aprB2*+) and infection with virulent *D. nodosus* in the generalized linear mixed model.

Variable	Trait	Estimate	SE	Z-value	p-value
Farm (region)	Presence of *D. nodosus*	5.28	0.69	7.63	< 0.0001
	Presence of virulent *D. nodosus*	5.32	0.70	7.55	< 0.0001
Residual		1.00	0.01	67.32	< 0.0001

#### Interbreed differences

The breeds with the highest risk of severe clinical footrot (OR > 10) were Swifter, Bentheim, all four Merino breeds (German Mutton Merino, German Merino-Mix, German Merino, Merino Longwool), Texel and German White Heath with Romney as reference breed (Table S5). The breeds with the lowest risk of developing clinical footrot include Romney, Ile-de-France, Suffolk and Pomeranian Coarsewool.

The mixed model estimates for the risk of infection with *D. nodosus* between Romney and all other breeds as well as between Swifter and all other breeds are shown as odds ratios (OR) in Tables 4 and S6–S7. Compared to Romney, all other breeds are at a higher risk of infection with *D. nodosus* and virulent *D. nodosus* (Table 4) (OR < 1). Romney sheep were less infected with *D. nodosus* than the other breeds investigated. With exception of Merino Longwool, all differences were significant. Compared to Swifter, all other breeds had a lower risk of infection with *D. nodosus* (Table S6) and virulent *D. nodosus* (Table S7) with all p-values being < 0.05. The corresponding OR for German White Heath, German Grey Heath, White Polled Heath, Merino Longwool, German Mutton Merino, German Merino, German Blackheaded Mutton, Suffolk and Texel can be found in Tables S8–S25. Comparing the local breeds German Grey Heath (GGH), German White Heath (GWH) and White Polled Heath (WPH), the GWH has a significantly higher risk of infection with *D. nodosus* (p = 0.0205; OR > 1) than WPH (Table S8). There is no significant difference when comparing GGH with GWH (Tables S10 and S11). However, GGH have a significantly higher risk of infection with virulent *D. nodosus* than WPH (Table S1). Our results show that the German Merino has a significantly higher risk of infection with *D. nodosus* compared to Ile-de-France, Texel and the German Whiteheaded Mutton (Tables S18–S19). On the other hand, the German Blackheaded Mutton has a significantly lower risk of infection with *D. nodosus* than Texel (Table S20) and a significantly lower risk of infection with virulent *D. nodosus* than German Whiteheaded Mutton (Table S21). Suffolk has a significantly higher risk of infection than Texel (Table S22). Furthermore, the risk of infection with virulent *D. nodosus* is significantly higher in the Suffolk breed than in the German Whiteheaded Mutton breed (Table S23). In the Texel breed, the risk of infection with virulent *D. nodosus* is significantly lower compared to Bentheim, Charollais, German Merino-Mix, German Merino, German Blackheaded Mutton, Suffolk, German Grey Heath and the German White Heath (Table S25).

**Table 4:** Odds ratios (OR) with their 95% confidence intervals (95%-CI) and p-values of Romney sheep compared to all other sheep breeds for risk to infection with *D. nodosus* (*aprV2*+*/aprB2*+) and virulent *D. nodosus* (*aprV2*+).

Breed of sheep	p-value	OR	95%-CI		p-value	OR	95%-CI	
	Risk of infection with all *D. nodosus*				Risk of infection with virulent *D. nodosus* only			
Bentheim	0.0023	0.053	0.008	0.349	0.0008	0.052	0.009	0.296
Charollais	0.0004	0.023	0.003	0.189	0.0035	0.060	0.009	0.398
Dorper	0.0027	0.039	0.005	0.325	0.0361	0.107	0.013	0.865
Coburg	0.0005	0.043	0.007	0.256	0.0023	0.096	0.021	0.433
Ile-de-France	0.0009	0.060	0.011	0.317	0.0063	0.149	0.038	0.584
Leine	0.0004	0.040	0.007	0.233	0.0055	0.118	0.026	0.533
German Mutton Merino	0.0106	0.085	0.013	0.563	0.0720	0.224	0.044	1.143
German Merino-Mix	< 0.0001	0.018	0.003	0.101	< 0.0001	0.038	0.009	0.163
German Merino	< 0.0001	0.033	0.006	0.172	0.0002	0.078	0.020	0.301
Merino Longwoll	0.1097	0.122	0.009	1.608	0.3113	0.324	0.037	2.867
White Polled Heath	0.0349	0.094	0.010	0.846	0.1888	0.295	0.048	1.824
East Friesian	0.0242	0.059	0.005	0.690	0.0512	0.087	0.007	1.013
German Blackheaded Mutton	0.0002	0.043	0.008	0.228	0.0015	0.111	0.029	0.431
Suffolk	0.0002	0.041	0.007	0.224	0.0013	0.097	0.023	0.402
Swifter	< 0.0001	< 0.001	< 0.001	< 0.001	< 0.0001	< 0.001	< 0.001	0.001
Texel	0.0030	0.079	0.015	0.423	0.0276	0.211	0.053	0.842
German Whiteheaded Mutton	0.0200	0.108	0.017	0.705	0.5481	0.583	0.100	3.392
Others	0.0010	0.067	0.013	0.336	0.0069	0.169	0.047	0.614
German Grey Heath	0.0003	0.031	0.005	0.201	0.0010	0.066	0.013	0.334
Pomeranian Coarsewool	0.0005	0.036	0.005	0.230	0.0032	0.089	0.018	0.445
German White Heath	< 0.0001	0.012	0.002	0.082	< 0.0001	0.025	0.005	0.134

OR < 1 indicate lower risk of infection with *D. nodosus* and virulent *D. nodosus* in Romney sheep compared to the other sheep breeds.

## Discussion

On the basis of the results of our previous report^26^, which revealed a high prevalence of *D. nodosus* in German sheep flocks, this follow-up study was conducted. The real-time PCR applied is a sensitive and specific method for the detection and discrimination of virulotypes of *D. nodosus* strains^15^. The combination of real-time PCR results of more than 9000 interdigital swab samples of sheep and personal interviews of sheep farmers may give new insights to evaluate possible risk factors for the infection of sheep with *D. nodosus* in general and virulent *D. nodosus* in particular. As many flocks were included in the study and the average sample size per flock was 44.7, risk factors were assessed in animals in mixed models accounting for a random farm effect. Therefore, estimates for fixed effects of risk factors should be unbiased by specific farm effects. The mostly small confidence intervals (Table 1) support the robustness of our results. It should be noted, that the design as a field study implies many levels of environmental conditions with unbalanced distributions. However, effect levels were not confounded allowing inferences independent of the other effects. The number of samples per breed was unevenly distributed but was mixed up with other fixed effects and not confined to single flocks. On average, one breed was represented in nine flocks. In 72 flocks, the sheep breeds overlapped, i.e. there was more than one breed in one and the same flock. This design allowed us to estimate breed effects that were not confounded by other effects under investigation in the present study. We tested for multicollinearity of the effects in the model using condition index and variance inflation factor. None of these diagnostic parameters indicated multicollinearity.

The highest prevalence of *D. nodosus* was detected in November to December. A similar high prevalence was observed in the months of January to February and May to June. The lowest prevalence was observed in September to October. Ovine footrot shows a seasonal pattern with more severe clinical signs and intensive transmission of *D. nodosus* during warm and humid environmental conditions^27^. In winter, most sheep in Germany are kept in stables where environmental conditions and high stocking rates in a confined space favour transmission of *D. nodosus* and the development of clinical signs. Gelasakis points out that the seasonal changes of footrot also depend on the production cycle^28^. In agreement with our results, Ardüser et al. reported in a Swiss study that the summer climate has a rather protective effect against *D. nodosus* infection, as does autumn compared to winter^17^. Angell et al. found an increase in the prevalence of footrot in late summer and spring^29^. This highlights the fact that the prevalence of *D. nodosus* in a flock and the manifestation of clinical disease do not strictly correlate throughout the year. These variations can be explained by the fact that at the onset of the disease, the highest burden of *D. nodosus* is found on feet with interdigital dermatitis (Scald)^30^ and that *D. nodosus* can persist in diseased feet^31^ over a long period of time.

Sheep from non-herdbook breeders had a significantly higher prevalence of *D. nodosus* than sheep from herdbook breeders. Herdbook breeders regularly attend shows and markets in order to sell breeding animals, and they have their sheep inspected by breed society staff. This may lead to greater attention being paid to the physical state and health of animals^32^, including claw health, and lameness is consequently treated at an early stage.

As expected, the percentage of infected sheep was higher if antibiotics had been used in the flock to treat footrot in the last 3–10 years, as this is an indication of the presence of the pathogen. Similarly, more animals suffered from footrot if there were clinical signs of footrot in the flock within the last 12 months prior to sampling. In flocks where footrot had occurred in the past, antibiotic treatment did probably not eliminate *D. nodosus* from the flock, but the pathogen persisted in the interdigital skin or in lesions within the hoof horn^33^. Thus, the exclusive application of antibiotics as the only measure to eradicate *D. nodosus* is not sufficient, but additional treatments must be applied. Similarly, another study showed that the factor “ever had footrot in herd” was a significant risk factor for infection with virulent *D. nodosus*^17^.

On the other hand, sheep tested more frequently negative for *D. nodosus* if they were treated with footbaths in the previous 3–10 years. This is plausible considering that footbaths have been proven to be effective in reducing or even eradicating *D. nodosus* in sheep flocks^16,19,34^. Weekly footbaths with a zinc sulfate disinfectant solution as the primary treatment of sheep flocks was shown to eliminate virulent *D. nodosus* in a Swiss study^19^. Footbathing to prevent interdigital dermatitis was associated with a lower risk of lameness^35^. The presence of other species like goats, cattle, horses and donkeys on the farm had no significant effect on the presence of *D. nodosus* on the feet of sheep in the present study. Transmission of different strains of *D. nodosus* between sheep and goats is possible on pasture plots^6^. Furthermore, the cross-infection of virulent *D. nodosus* between sheep and co-grazing cattle has been reported in a previous study^4^. While the “contact on pasture to goats” had a significant effect in a Swiss study, it was also found to be a confounding factor, as no goats were infected with virulent *D. nodosus*^17^. Interestingly, in the final multivariable model, the sex of the animal had a significant effect only on the presence of virulent *D. nodosus*, while no significant effect on the presence of *D. nodosus* was found in general. In agreement with our results, sex was not a risk factor associated with the infection with *D. nodosus* in the study conducted by Ardüser et al.^17^. However, non-genetic effects on the susceptibility to footrot, including sex were reported previously, with higher footrot scores found in male lambs than in females^12,36^. In contrast to other studies, the factor "age of the animal" had no significant effect on the status of infection with *D. nodosus*. Two studies showed that yearlings were much less likely to have footrot than lambs or adults^29,34^ and similarly, adults showed a higher prevalence of virulent *D. nodosus* than yearlings and lambs^17^. Further, Nieuwhof et al. found that the risk of clinical signs of footrot increases with body weight of the animal and to a lesser extent with age, suggesting that the risk is higher in fast growing lambs^37^.

It has long been shown that the efficiency of the commercially available multivalent vaccine against footrot can be low due to antigenic competition^38^. Vaccination of sheep had no favourable effect on the prevalence of footrot in a flock^39^. Allworth et al. reported that the whole-cell vaccine was only 33% effective compared to the recombinant vaccine (46%) and thus, suggesting footbathing as the superior treatment strategy against footrot compared to vaccination (97% and 91% effective with weekly or 3-weekly footbaths, respectively)^34^. This is consistent with our results, as we were not able to show significant benefits of vaccination reported within 12 months or in the previous 3–10 years in terms of reducing the incidence of *D. nodosus*. Nevertheless, Kraft et al. found that the combination of repeated individual treatments with antimicrobials and commercial vaccine prior to challenge periods was also successful in eradicating *D. nodosus* from sheep flocks^16^. In another study, a flock-specific vaccine may have helped eradicate virulent footrot in migratory sheep flocks in Nepal^40^. According to Winter et al., vaccination of ewes once per year was associated with a lower risk of lameness in the flock^35^. Fewer new infections and faster recovery rate from footrot lesions were found in vaccinated sheep compared to unvaccinated sheep^41^ and genetic variation affects the immune response to vaccination^42^. More specifically, vaccine protection lasted longer in experimental footrot infection in Romneys than in Merinos ^23^.

Genetic variation for footrot resistance involves inter-breed differences, within-breed variation and responses to vaccinations. Within-breed variation has been studied for breeds such as Romney Marsh and Corriedale by Skerman and Moorhouse^43^ who claimed to have bred bloodlines with increased resistance to footrot through deliberate field challenge. Patterson and Patterson aimed at breeding high performing Merinos with an increased resistance to footrot through selective breeding and culling affected ewes^44^. Furthermore, Mucha et al. detected seven on the chromosome-level significant SNPs in a genome-wide association study of footrot in Texel sheep^24^. New Zealand’s breeding programme for footrot resistance is based on the hypothesis that footrot resistance is present in all types of fine-wool sheep^45^. Inter-breed variation is rarely studied because breed differences in response to footrot are often confounded in studies, if sheep are not exposed to the same challenge conditions^46^. In our field study design, we employed statistical methods to compare the different sheep breeds on farms accounting for farm, region and seasonal effects as well as for other significant risk factors in multivariable generalized mixed models. Our design allowed unbiased estimates for breed effects because sheep breeds were not confounded with one specific herd and other risk factors due to the wide variation of effect levels by breeds. A study by Nieuwhof et al. comparing Scottish Blackface and Mules^37^ showed some inter-breed variation. Emery et al. compared the susceptibility of five sheep breeds to footrot (Romney Marsh, Dorset Horn, Border Leicester, Peppin Merinos, Saxon Merinos) and concluded that British breeds seem to be more resistant to footrot than wool sheep such as Merinos^22^. Our findings confirm that Romney sheep have the lowest risk for severe footrot scores and infection with *D. nodosus* compared to more than 20 other breeds. On the contrary, Emery et al. also stated that this resistance in British breeds manifests as a rapid resolution of benign foot lesions and not as reduction in the number of feet affected. An explanation for these results conflicting our study could be the different detection methods (clinical assessment of foot lesions vs. qPCR in our study). In addition, the Emery-study was designed as an infection trial, while we performed a field study. Although findings about the risk for Swifter should be interpreted with caution with regard to the low number of flocks, the results indicate an increased risk of infection with *D. nodosus* and severe footrot signs for this breed. Swifter is a cross-breed between Texel and Flemish milk sheep, specifically bred to increase the productivity of the sheep population in the Netherlands. To the best of our knowledge, we provide the first information about the risk of infection and footrot signs for Swifter and the mostly indigenous local breeds German Grey Heath, German White Heath and White Polled Heath. The results shed light on the extensive variability between breeds. Further work is required to evaluate the risks of further breeds and help understand the causes of these differences.

Our results provide new insights on the issue of interbreed variation in resistance to ovine footrot. All risk factors were evaluated under consideration of farm and other significant effects in order to avoid confounding with breed effects. The greatest influence on the risk of an animal becoming infected with *D. nodosus* in general and virulent *D. nodosus* in particular was the individual farm and thus its management practices and environmental conditions. In total, 84% of the variability between flocks is individual and herd specific. It remains unknown which particular factors contribute to the dynamics of *D. nodosus* within the individual sheep flocks and further research is required to determine these factors. Besides, the breed has a significant influence on the risk of animals becoming infected with *D. nodosus*. In agreement with a previous study, we propose that Romney sheep had the lowest risk of infection with *D. nodosus* and virulent *D. nodosus* in particular, compared to more than 20 breeds. Further, Swifter had the highest risk of infection, and White Polled Heath had a significantly lower risk compared to German Grey Heath and German White Heath.

## Methods

### Ethical approval

The study was conducted according to the guidelines of the Declaration of Helsinki, and approved by the Institutional Review Board of the University of Veterinary Medicine Hannover (Foundation) and the state veterinary offices from the different German Federal States with the following registration numbers for Lower Saxony 33.19-42502-05-19A414 (8 April 2019), Schleswig–Holstein V 244-25573/2019 (20 May 2019), North Rhine-Westphalia 81-02.05.40.19.041 (28 May 2019), Brandenburg 2347-A-19-1-2019 (18 June 2019), Saxony DD24.1-5131/475/4 (20 May 2019), Saxony-Anhalt 42502-3-862 (01 July 2019), Thuringia 33.19-42502-05-19A414 (11 June 2019), Bavaria 55.2-2532.Vet_03-19-29 (20 May 2019), 55.2.2-2532.2-943-6 (03 June 2019), Hesse V54-19 c 20/15-V/Anz.1024 (21 May 2019) and RPKS-23-19 c 16/1-2019/1 (09 May 2019). We confirm that the reporting of the study follows the recommendations in the ARRIVE guidelines.

### Study design

We conducted a cross-sectional follow-up study to evaluate potential risk factors that are associated with the infection of sheep with *D. nodosus*, specifically the virulent strain of *D. nodosus* and the footrot score. All farmers recruited for our previous study on prevalence of footrot and *D. nodosus* participated voluntarily and gave written informed consent for participating in further research on risk factors. An additional farm agreed to collect data, so we got in total samples from 208 flocks. On each of the 208 farms, the veterinarians conducted an interview with the sheep farmer during the visit on the day of sampling. The answers were documented on a written questionnaire (Table S26). The interview contained yes–no and closed questions on herdbook membership, flock size, sheep breeds, other livestock species present on the farm, reports of previous outbreaks of footrot and strategies for treatment of diseased animals. Depending on the location of the farm, each flock was assigned to one of three study areas (Northern Germany, Eastern Germany and Southern Germany). Samples were collected from January 2019 until September 2020 and sampling of animals within a flock was randomized.

Flock sizes ranged from 10 to 2400 with a mean sample size per flock of 44.7. The number of breeds per flock was 1–7, with 136 flocks keeping one breed and 72 flocks keeping 2–7 breeds (Table S27). The breeds included in the analysis were distributed across 2–45 flocks with a median number of nine flocks (Table S28). Breeds with less than 50 animals or that were not represented in more than two flocks were grouped under "other breeds". We set this limit to exclude breeds with very small numbers of individuals in several flocks and to avoid confounding effects in the statistical model. The only breed included and kept in just two different flocks were Swifter sheep. The reason for this exemption was that we wanted to compare their susceptibility with that of all other breeds. Complete flock sampling was done on 60 farms. One interdigital swab was taken from all four feet per sheep.

For the DNA extraction from the cotton swabs, the Qiagen DNeasy Blood and Tissue Kit (Qiagen, Hilden, Germany) was used according to the manufacturer’s protocol. The DNA samples were tested using real-time PCR to discriminate virulent and benign *D. nodosus* strains based on the aprV2/aprB2 gene polymorphisms following the protocol of Stäuble et al.^15^ The 25-µL reaction mixture containing 22.5 µL TaqMan Fast Advanced MasterMix (Life Technologies, Thermo Fischer Scientific, Waltham, MA, USA) and 2.5 µL of the extracted sample DNA were pipetted in duplicates into a 96-well plate. Two non-template samples (pyrogen-free water) were used as negative controls for each qPCR run. The positive controls were DNA of the virulent type strain ATCC 25549^T^ and the benign field isolate JF5922 provided by the Institute of Veterinary Bacteriology, University of Bern, Bern, Switzerland^48^. The amplification was carried out in a 7500 Real-Time PCR System (ABI, Thermo Fischer Scientific, Waltham, MA, USA). For the interpretation of the results, we used QuantStudio 3 System Software (Thermo Fisher Scientific, Waltham, MA, USA) with the threshold set at 0.065. The threshold of the Ct-value for a positive sample was set at < 40.

### Statistical analysis

All factors were first tested in univariable models employing generalized mixed linear models for the binomial traits "presence of *D. nodosus* or virulent *D. nodosus"* and the categorical trait "footrot score" with 6 classes from 0 to 5 according to the Footrot Scoring System of the Swiss Consulting and Health Service for Small Ruminants^47^ for individual sheep. In the generalized mixed linear model, we employed a binomial distribution function and logit as link function for binary variates and for categorical variates a multinomial distribution function with a cumulative logit link function to parameterize the probability of *D. nodosus* infection and the footrot score in individual animals.

All factors with p-values < 0.05 were retained for further analysis in a backward elimination strategy. The final model was validated through a basic model with the effects of region, farm within region, month of recording and herdbook member, two further extended basic models with the additional effects of year of recording, sex of the animal, and age of the animal with a forward elimination for each one additional effect. The final generalized mixed linear model using logits for probabilities of *D. nodosus* infection and footrot scores in individual animals (*θ*_*ijklmnopqr*_) was as follows:

$$\theta_{ijklmnopqr} = {\text{ log}}\left[ {p_{ijklmnopqr} /\left( {{1}\, - \,p_{ijklmnopqr} } \right)} \right] \, = \mu \, + \,{\text{region}}_{{\text{i}}} + {\text{ farm}}\left( {{\text{region}}} \right)_{{{\text{ij}}}} + {\text{ month}}_{{\text{k}}} + {\text{ herdbook}}_{l} \, + {\text{ year}}_{m} + {\text{ sex}}_{{\text{n}}} + {\text{ breed}}_{{\text{o}}} + {\text{ treatab}}_{{\text{p}}} + {\text{ treatfb}}_{{\text{q}}} + {\text{ clinfrt}}_{{\text{r}}} + {\text{ e}}_{ijklmnopqr} ,$$

where *μ* is an unknown constant common to all sheep, region_i_ = fixed of the region, where the flock is located, with i = northern, southern and eastern Germany; farm(region)_ij_ = random effect of the farm_j_ within region_i_ for 208 flocks; month_k_ = fixed effect of two-month-classes when data were recorded, with k = Januar–February, March–April, May–June, July–August, September–October, November–December; herdbook_*l*_ = fixed effect of the herdbook with l = yes or no; year_*m*_ = fixed effect of the year with m = 2019 or 2020; sex_n_ = fixed effect of the sex of the animal with n = male or female; breed_o_ = fixed effect of the breed with o = Romney, Bentheim, Charollais, Dorper, Coburg, Ile-de-France, Leine, German Mutton Merino, German Merino-Mix, German Merino, Merino Longwool, White Polled Heath, East Friesian, German Blackheaded Mutton, Suffolk, Swifter, Texel, German Whiteheaded Mutton, German Grey Heath, Pomeranian Coarsewool, German White Heath or others; treatab_p_ = fixed effect of treatment of diseased sheep with antibiotics in the past 3–10 years with p = yes, no or no response (Antibiotics, 3–10); treatfb_q_ = fixed effect of treatment of diseased sheep with footbaths in the past 3–10 years with q = yes, no or no response (Footbaths, 3–10); clinfrt_r_ = fixed effect of clinical signs of footrot in the flock within the past 12 months with r = yes, no or no response (Footrot, 12); e_*ijklmnopqr*_ an unknown random residual effect. All breeds included in the respective model, except for Swifter, were present in more than two different sheep flocks.

Tables S2–S4 show the model number (1–11) and which factors were added to the basic model in a forward selection method. In model 1, the factors year of recording and sex of the animal were added to the basic model. In model 2, the factor age of the animal was added to the basic model. The factors with p-values < 0.05 were then tested in the final multivariable generalized mixed linear model. Backward selection was performed to test the consistency of the results. For the analysis of the footrot scores, a generalized mixed linear model with an ordered multinomial distribution function and logit as link function was performed (Table S4). In the final model, a p-value < 0.05 was defined as the significance threshold for inclusion of an effect (Table 2).

Risk factor analysis for the infection with *D. nodosus*, virulent *D. nodosus* and footrot scores on individual sheep was performed using SAS, version 9.4 (Statistical Analysis System, Cary, NC, USA, 2021). Because of the large sample size per flock with a mean of 44.7 and a large variation of the within-flock prevalence from 0 to 100%, the farm was considered as random effect in the analyses and all risk factors were analysed in animals. We used SAS for testing multicollinearity of the effects regarded in the model. Diagnostic parameters calculated were condition index and variance inflation factor. Condition indices larger than 10 or variance inflation factors larger than 5 were set as thresholds for the presence of multicollinearity.

## Supplementary Information

Supplementary Tables.

## Data Availability

All necessary information needed to support the results can be found in the manuscript or are available from the corresponding author on reasonable request and with the permission of the sheep farmers.
