# Supplementary material for: Risk factors associated with the infection of sheep with Dichelobacter nodosus
Source: Sci Rep. 2022 Jun 15;12:10032. doi: 10.1038/s41598-022-13933-4 (PMC9200780; doi:10.1038/s41598-022-13933-4)
Supplement: Supplementary file 1 — Supplementary Tables. [file 41598_2022_13933_MOESM1_ESM.docx]

**Table S1.** Real-time PCR results of 9,297 interdigital swab samples collected in 208 German sheep flocks

| **Real-time PCR result** | **Number of sheep** | **Frequency (%)** |
| --- | --- | --- |
|  |  |  |
| Benign (*aprB2+*) | 188 | 2.02 |
| Virulent and benign (*aprV2+/aprB2+)* | 195 | 2.10 |
| Virulent (*aprV2+*) | 5328 | 57.31 |
| Free of *D. nodosus* | 3586 | 38.57 |
| Total | 9297 | 100.00 |

**Table S2.** Model numbers, numbers (No.) and frequencies (%) of animals, degrees of freedom (DF), F-values and p-values of the univariable models employing generalized mixed linear models for the trait presence of *D. nodosus*. The basic model contained the effects region, farm within region, month of recording and herdbook member. All further models numbered from 1 to 11 include the effects of the basic model.

| **Model number** | **Source of variation** | **Category** | **No. of animals**  **N %** | | **DF** | **F-value** | **P-value** |
| --- | --- | --- | --- | --- | --- | --- | --- |
| **Basic model** | **Region** |  |  |  | 2 | 0.99 | 0.3726 |
|  |  | Northern Germany | 4238 | 45.58 |  |  |  |
|  |  | Eastern Germany | 3463 | 37.25 |  |  |  |
|  |  | Southern Germany | 1596 | 17.17 |  |  |  |
| **Basic model** | **Month of recording** |  |  |  | 5 | 12.31 | <0.0001 |
|  |  | 1 (Jan-Feb) | 1204 | 12.95 |  |  |  |
|  |  | 2 (Mar-Apr) | 1858 | 19.98 |  |  |  |
|  |  | 3 (May-Jun) | 1898 | 20.42 |  |  |  |
|  |  | 4 (Jul-Aug) | 999 | 10.75 |  |  |  |
|  |  | 5 (Sep-Oct) | 1399 | 15.05 |  |  |  |
|  |  | 6 (Nov-Dec) | 1939 | 20.86 |  |  |  |
| **Basic model** | **Herdbook member** |  |  |  | 1 | 5.78 | 0.0162 |
|  |  | Yes | 4885 | 52.54 |  |  |  |
|  |  | No | 4412 | 47.46 |  |  |  |
| **1** | **Year** |  |  |  | 1 | 4.66 | 0.1990 |
|  |  | 2019 | 3369 | 36.24 |  |  |  |
|  |  | 2020 | 5928 | 63.76 |  |  |  |
| **1** | **Sex of the animal** |  |  |  | 1 | 1.39 | 0.2377 |
|  |  | Male | 1375 | 14.79 |  |  |  |
|  |  | Female | 7922 | 85.21 |  |  |  |
| **2** | **Age of the animal** |  |  |  | 1 | 0.09 | 0.7598 |
|  |  | Lamb (<1 year) | 843 | 9.07 |  |  |  |
|  |  | Sheep (≥1 year) | 8454 | 90.93 |  |  |  |
| **3** | **Breed** |  |  |  | 21 | 6.70 | <0.0001 |
|  |  | Romney | 80 | 0.86 |  |  |  |
|  |  | Bentheim | 93 | 1.00 |  |  |  |
|  |  | Charollais | 74 | 0.80 |  |  |  |
|  |  | Dorper | 279 | 3.00 |  |  |  |
|  |  | Coburg | 125 | 1.34 |  |  |  |
|  |  | Ile-De-France | 212 | 2.28 |  |  |  |
|  |  | Leine | 513 | 5.52 |  |  |  |
|  |  | German Mutton Merino | 156 | 1.68 |  |  |  |
|  |  | German Merino-Mix | 730 | 7.85 |  |  |  |
|  |  | German Merino | 1311 | 14.10 |  |  |  |
|  |  | Merino Longwool | 251 | 2.70 |  |  |  |
|  |  | White Polled Heath | 192 | 2.07 |  |  |  |
|  |  | East Friesian | 230 | 2.47 |  |  |  |
|  |  | German Blackheaded Mutton | 681 | 7.32 |  |  |  |
|  |  | Suffolk | 725 | 7.80 |  |  |  |
|  |  | Swifter | 147 | 1.58 |  |  |  |
|  |  | Texel | 679 | 7.30 |  |  |  |
|  |  | German Whiteheaded Mutton | 224 | 2.41 |  |  |  |
|  |  | German Grey Heath | 334 | 3.59 |  |  |  |
|  |  | Pomeranian Coarsewool | 98 | 1.05 |  |  |  |
|  |  | German White Heath | 178 | 1.91 |  |  |  |
|  |  | Others | 1985 | 21.35 |  |  |  |
| **4** | **Flock size** |  |  |  | 1 | 0.28 | 0.5958 |
| **4** | **Number of rams on the farm** |  |  |  | 1 | 0.11 | 0.7354 |
| **5** | **Goats on the farm** |  |  |  | 2 | 0.35 | 0.7019 |
|  |  | Yes | 2376 | 25.56 |  |  |  |
|  |  | No | 5320 | 57.22 |  |  |  |
|  |  | No response | 1601 | 17.22 |  |  |  |
| **6** | **Cattle on the farm** |  |  |  | 2 | 0.25 | 0.7804 |
|  |  | Yes | 2022 | 21.75 |  |  |  |
|  |  | No | 5320 | 61.03 |  |  |  |
|  |  | No response | 1601 | 17.22 |  |  |  |
| **7** | **Donkeys on the farm** |  |  |  | 2 | 0.20 | 0.8226 |
|  |  | Yes | 1530 | 16.46 |  |  |  |
|  |  | No | 6166 | 66.32 |  |  |  |
|  |  | No response | 1601 | 17.22 |  |  |  |
| **8** | **Horses on the farm** |  |  |  | 2 | 0.05 | 0.9560 |
|  |  | Yes | 1318 | 14.18 |  |  |  |
|  |  | No | 6376 | 68.58 |  |  |  |
|  |  | No response | 1603 | 17.24 |  |  |  |
| **9** | **Treatment of diseased sheep with antibiotics for footrot within the last 12 months** |  |  |  | 2 | 1.40 | 0.2478 |
|  |  | Yes | 2245 | 48.78 |  |  |  |
|  |  | No | 5489 | 35.99 |  |  |  |
|  |  | No response | 1563 | 15.23 |  |  |  |
| **9** | **Treatment of sheep with footbaths within the last 12 months** |  |  |  | 2 | 0.33 | 0.7188 |
|  |  | Yes | 3000 | 32.27 |  |  |  |
|  |  | No | 4760 | 51.20 |  |  |  |
|  |  | No response | 1537 | 16.53 |  |  |  |
| **9** | **Treatment of sheep with a vaccination against footrot within the last 12 months** |  |  |  | 2 | 0.33 | 0.7212 |
|  |  | Yes | 2322 | 24.98 |  |  |  |
|  |  | No | 5415 | 58.24 |  |  |  |
|  |  | No response | 1560 | 16.78 |  |  |  |
| **10** | **Treatment of diseased sheep with antibiotics in the previous 3-10 years** |  |  |  | 2 | 3.21 | 0.0402 |
|  |  | Yes | 495 | 5.32 |  |  |  |
|  |  | No | 237 | 2.55 |  |  |  |
|  |  | No response | 8565 | 92.13 |  |  |  |
| **10** | **Treatment of sheep with footbaths in the previous 3-10 years** |  |  |  | 2 | 6.43 | 0.0016 |
|  |  | Yes | 276 | 2.97 |  |  |  |
|  |  | No | 462 | 4.97 |  |  |  |
|  |  | No response | 8559 | 92.06 |  |  |  |
| **10** | **Treatment of sheep with a vaccination against footrot in the previous 3-10 years** |  |  |  | 2 | 0.42 | 0.6582 |
|  |  | Yes | 364 | 3.92 |  |  |  |
|  |  | No | 506 | 5.44 |  |  |  |
|  |  | No response | 8427 | 90.64 |  |  |  |
| **11** | **Clinical signs of footrot on animals in the flock within the last 12 months** |  |  |  | 2 | 4.90 | 0.0074 |
|  |  | Yes | 4535 | 48.78 |  |  |  |
|  |  | No | 3346 | 35.99 |  |  |  |
|  |  | No response | 1416 | 15.23 |  |  |  |
| **11** | **Clinical signs of footrot in the flock in the previous 3-10 years** |  |  |  | 2 | 0.89 | 0.4103 |
|  |  | Yes | 6155 | 66.20 |  |  |  |
|  |  | No | 1316 | 14.16 |  |  |  |
|  |  | No response | 1826 | 19.64 |  |  |  |

**Table S3.** Model numbers, numbers (No.) and frequencies (%) of animals, degrees of freedom (DF), F-values and p-values of the univariable models employing generalized mixed linear models for the trait infection with virulent *D. nodosus*. The basic model contained the effects region, farm within region, month of recording and herdbook member. All further models numbered from 1 to 11 included the effects of the basic model.

| **Model number** | **Source of variation** | **Category** | **No. of animals**  **N %** | | **DF** | **F-value** | **P-value** |
| --- | --- | --- | --- | --- | --- | --- | --- |
| **Basic model** | **Region** |  |  |  | 2 | 1.86 | 0.1589 |
|  |  | Northern Germany | 4238 | 45.58 |  |  |  |
|  |  | Eastern Germany | 3463 | 37.25 |  |  |  |
|  |  | Southern Germany | 1596 | 17.17 |  |  |  |
| **Basic model** | **Month of recording** |  |  |  | 5 | 4.18 | 0.0009 |
|  |  | 1 (Jan-Feb) | 1204 | 12.95 |  |  |  |
|  |  | 2 (Mar-Apr) | 1858 | 19.98 |  |  |  |
|  |  | 3 (May-Jun) | 1898 | 20.42 |  |  |  |
|  |  | 4 (Jul-Aug) | 999 | 10.75 |  |  |  |
|  |  | 5 (Sep-Oct) | 1399 | 15.05 |  |  |  |
|  |  | 6 (Nov-Dec) | 1939 | 20.86 |  |  |  |
| **Basic model** | **Herdbook member** |  |  |  | 1 | 14.06 | 0.0002 |
|  |  | Yes | 4885 | 52.54 |  |  |  |
|  |  | No | 4412 | 47.46 |  |  |  |
| **1** | **Year** |  |  |  | 1 | 3.84 | 0.0501 |
|  |  | 2019 | 3369 | 36.24 |  |  |  |
|  |  | 2020 | 5928 | 63.76 |  |  |  |
| **1** | **Sex of the animal** |  |  |  | 1 | 4.56 | 0.0327 |
|  |  | Male | 1375 | 14.79 |  |  |  |
|  |  | Female | 7922 | 85.21 |  |  |  |
| **2** | **Age of the animal** |  |  |  | 1 | 0.32 | 0.5693 |
|  |  | Lamb (<1 year) | 843 | 9.07 |  |  |  |
|  |  | Sheep (>1 year) | 8454 | 90.93 |  |  |  |
| **3** | **Breed** |  |  |  | 21 | 7.25 | <0.0001 |
|  |  | Romney | 80 | 0.86 |  |  |  |
|  |  | Bentheim | 93 | 1.00 |  |  |  |
|  |  | Charollais | 74 | 0.80 |  |  |  |
|  |  | Dorper | 279 | 3.00 |  |  |  |
|  |  | Coburg | 125 | 1.34 |  |  |  |
|  |  | Ile-De-France | 212 | 2.28 |  |  |  |
|  |  | Leine | 513 | 5.52 |  |  |  |
|  |  | German Mutton Merino | 156 | 1.68 |  |  |  |
|  |  | German Merino-Mix | 730 | 7.85 |  |  |  |
|  |  | German Merino | 1311 | 14.10 |  |  |  |
|  |  | Merino Longwool | 251 | 2.70 |  |  |  |
|  |  | White Polled Heath | 192 | 2.07 |  |  |  |
|  |  | East Friesian | 230 | 2.47 |  |  |  |
|  |  | German Blackheaded Mutton | 681 | 7.32 |  |  |  |
|  |  | Suffolk | 725 | 7.80 |  |  |  |
|  |  | Swifter | 147 | 1.58 |  |  |  |
|  |  | Texel | 679 | 7.30 |  |  |  |
|  |  | German Whiteheaded Mutton | 224 | 2.41 |  |  |  |
|  |  | German Grey Heath | 334 | 3.59 |  |  |  |
|  |  | Pomeranian Coarsewool | 98 | 1.05 |  |  |  |
|  |  | German White Heath | 178 | 1.91 |  |  |  |
|  |  | Others | 1985 | 21.35 |  |  |  |
| **4** | **Flock size** |  |  |  | 1 | 0.20 | 0.6516 |
| **4** | **Number of rams on the farm** |  |  |  | 1 | 0.01 | 0.9195 |
| **5** | **Goats on the farm** |  |  |  | 2 | 0.32 | 0.7278 |
|  |  | Yes | 2376 | 25.56 |  |  |  |
|  |  | No | 5320 | 57.22 |  |  |  |
|  |  | No response | 1601 | 17.22 |  |  |  |
| **6** | **Cattle on the farm** |  |  |  | 2 | 0.14 | 0.8696 |
|  |  | Yes | 2022 | 21.75 |  |  |  |
|  |  | No | 5320 | 61.03 |  |  |  |
|  |  | No response | 1601 | 17.22 |  |  |  |
| **7** | **Donkeys on the farm** |  |  |  | 2 | 0.03 | 0.9718 |
|  |  | Yes | 1530 | 16.46 |  |  |  |
|  |  | No | 6166 | 66.32 |  |  |  |
|  |  | No response | 1601 | 17.22 |  |  |  |
| **8** | **Horses on the farm** |  |  |  | 2 | 0.22 | 0.8007 |
|  |  | Yes | 1318 | 14.18 |  |  |  |
|  |  | No | 6376 | 68.58 |  |  |  |
|  |  | No response | 1603 | 17.24 |  |  |  |
| **9** | **Treatment of diseased sheep with antibiotics for footrot within the last 12 months** |  |  |  | 2 | 2.02 | 0.1326 |
|  |  | Yes | 2245 | 48.78 |  |  |  |
|  |  | No | 5489 | 35.99 |  |  |  |
|  |  | No response | 1563 | 15.23 |  |  |  |
| **9** | **Treatment of sheep with footbaths within the last 12 months** |  |  |  | 2 | 0.50 | 0.6036 |
|  |  | Yes | 3000 | 32.27 |  |  |  |
|  |  | No | 4760 | 51.20 |  |  |  |
|  |  | No response | 1537 | 16.53 |  |  |  |
| **9** | **Treatment of sheep with a vaccination against footrot within the last 12 months** |  |  |  | 2 | 0.25 | 0.7800 |
|  |  | Yes | 2322 | 24.98 |  |  |  |
|  |  | No | 5415 | 58.24 |  |  |  |
|  |  | No response | 1560 | 16.78 |  |  |  |
| **10** | **Treatment of diseased sheep with antibiotics in the previous 3-10 years** |  |  |  | 2 | 3.31 | 0.0366 |
|  |  | Yes | 495 | 5.32 |  |  |  |
|  |  | No | 237 | 2.55 |  |  |  |
|  |  | No response | 8565 | 92.13 |  |  |  |
| **10** | **Treatment of sheep with footbaths in the previous 3-10 years** |  |  |  | 2 | 4.12 | 0.0162 |
|  |  | Yes | 276 | 2.97 |  |  |  |
|  |  | No | 462 | 4.97 |  |  |  |
|  |  | No response | 8559 | 92.06 |  |  |  |
| **10** | **Treatment of sheep with a vaccination against footrot in the previous 3-10 years** |  |  |  | 2 | 0.15 | 0.8631 |
|  |  | Yes | 364 | 3.92 |  |  |  |
|  |  | No | 506 | 5.44 |  |  |  |
|  |  | No response | 8427 | 90.64 |  |  |  |
| **11** | **Clinical signs of footrot in the flock within the last 12 months** |  |  |  | 2 | 5.53 | 0.0040 |
|  |  | Yes | 4535 | 48.78 |  |  |  |
|  |  | No | 3346 | 35.99 |  |  |  |
|  |  | No response | 1416 | 15.23 |  |  |  |
| **11** | **Clinical signs of footrot in the flock in the previous 3-10 years** |  |  |  | 2 | 0.22 | 0.8047 |
|  |  | Yes | 6155 | 66.20 |  |  |  |
|  |  | No | 1316 | 14.16 |  |  |  |
|  |  | No response | 1826 | 19.64 |  |  |  |

**Table S4.** Model numbers, numbers (No.) and frequencies (%) of animals, degrees of freedom (DF), F-values and p-values of the categorical trait footrot score with 6 classes from 0 to 5 employing a generalized mixed linear model using an ordered multinomial distribution function and cumulative logits as link function. The basic model contained the effects region, farm within region, month of recording and herdbook member. All further models numbered from 1 to 10 included the effects of the basic model.

| **Model number** | **Source of variation** | **Category** | **No. of animals**  **N %** | | **DF** | **F-value** | **P-value** |
| --- | --- | --- | --- | --- | --- | --- | --- |
| **Basic model** | **Region** |  |  |  | 2 | 0.19 | 0.8247 |
|  |  | Northern Germany | 4238 | 45.58 |  |  |  |
|  |  | Eastern Germany | 3463 | 37.25 |  |  |  |
|  |  | Southern Germany | 1596 | 17.17 |  |  |  |
| **Basic model** | **Month of recording** |  |  |  | 5 | 3.29 | 0.0057 |
|  |  | 1 (Jan-Feb) | 1204 | 12.95 |  |  |  |
|  |  | 2 (Mar-Apr) | 1858 | 19.98 |  |  |  |
|  |  | 3 (May-Jun) | 1898 | 20.42 |  |  |  |
|  |  | 4 (Jul-Aug) | 999 | 10.75 |  |  |  |
|  |  | 5 (Sep-Oct) | 1399 | 15.05 |  |  |  |
|  |  | 6 (Nov-Dec) | 1939 | 20.86 |  |  |  |
| **Basic model** | **Herdbook member** |  |  |  | 1 | 14.29 | 0.0002 |
|  |  | Yes | 4885 | 52.54 |  |  |  |
|  |  | No | 4412 | 47.46 |  |  |  |
| **Basic model** | **Year** |  |  |  | 1 | 26.66 | <0.0001 |
|  |  | 2019 | 3369 | 36.24 |  |  |  |
|  |  | 2020 | 5928 | 63.76 |  |  |  |
| **Basic model** | **Sex of the animal** |  |  |  | 1 | 18.90 | <0.0001 |
|  |  | Male | 1375 | 14.79 |  |  |  |
|  |  | Female | 7922 | 85.21 |  |  |  |
| **1** | **Age of the animal** |  |  |  | 1 | 3.78 | 0.0519 |
|  |  | Lamb (<1 year) | 843 | 9.07 |  |  |  |
|  |  | Sheep (>1 year) | 8454 | 90.93 |  |  |  |
| **2** | **Flock size** |  |  |  | 1 | 0.01 | 0.9339 |
| **2** | **Number of rams on the farm** |  |  |  | 1 | 0.02 | 0.8850 |
| **3** | **Goats on the farm** |  |  |  | 2 | 2.49 | 0.0827 |
|  |  | Yes | 2376 | 25.56 |  |  |  |
|  |  | No | 5320 | 57.22 |  |  |  |
|  |  | No response | 1601 | 17.22 |  |  |  |
| **4** | **Cattle on the farm** |  |  |  | 2 | 2.06 | 0.1279 |
|  |  | Yes | 2022 | 21.75 |  |  |  |
|  |  | No | 5320 | 61.03 |  |  |  |
|  |  | No response | 1601 | 17.22 |  |  |  |
| **5** | **Donkeys on the farm** |  |  |  | 2 | 2.09 | 0.1237 |
|  |  | Yes | 1530 | 16.46 |  |  |  |
|  |  | No | 6166 | 66.32 |  |  |  |
|  |  | No response | 1601 | 17.22 |  |  |  |
| **6** | **Horses on the farm** |  |  |  | 2 | 3.77 | 0.0232 |
|  |  | Yes | 1318 | 14.18 |  |  |  |
|  |  | No | 6376 | 68.58 |  |  |  |
|  |  | No response | 1603 | 17.24 |  |  |  |
| **7** | **Treatment of diseased sheep with antibiotics for footrot within the last 12 months** |  |  |  | 2 | 0.61 | 0.5439 |
|  |  | Yes | 2245 | 48.78 |  |  |  |
|  |  | No | 5489 | 35.99 |  |  |  |
|  |  | No response | 1563 | 15.23 |  |  |  |
| **7** | **Treatment of sheep with footbaths within the last 12 months** |  |  |  | 2 | 0.43 | 0.6481 |
|  |  | Yes | 3000 | 32.27 |  |  |  |
|  |  | No | 4760 | 51.20 |  |  |  |
|  |  | No response | 1537 | 16.53 |  |  |  |
| **7** | **Treatment of sheep with a vaccination against footrot within the last 12 months** |  |  |  | 2 | 0.74 | 0.4763 |
|  |  | Yes | 2322 | 24.98 |  |  |  |
|  |  | No | 5415 | 58.24 |  |  |  |
|  |  | No response | 1560 | 16.78 |  |  |  |
| **8** | **Treatment of diseased sheep with antibiotics in the previous 3-10 years** |  |  |  | 2 | 1.47 | 0.2301 |
|  |  | Yes | 495 | 5.32 |  |  |  |
|  |  | No | 237 | 2.55 |  |  |  |
|  |  | No response | 8565 | 92.13 |  |  |  |
| **8** | **Treatment of sheep with footbaths in the previous 3-10 years** |  |  |  | 2 | 0.66 | 0.5183 |
|  |  | Yes | 276 | 2.97 |  |  |  |
|  |  | No | 462 | 4.97 |  |  |  |
|  |  | No response | 8559 | 92.06 |  |  |  |
| **8** | **Treatment of sheep with a vaccination against footrot in the previous 3-10 years** |  |  |  | 2 | 0.82 | 0.4410 |
|  |  | Yes | 364 | 3.92 |  |  |  |
|  |  | No | 506 | 5.44 |  |  |  |
|  |  | No response | 8427 | 90.64 |  |  |  |
| **9** | **Clinical signs of footrot in the flock within the last 12 months** |  |  |  | 2 | 3.25 | 0.0387 |
|  |  | Yes | 4535 | 48.78 |  |  |  |
|  |  | No | 3346 | 35.99 |  |  |  |
|  |  | No response | 1416 | 15.23 |  |  |  |
| **9** | **Clinical signs of footrot in the flock in the previous 3-10 years** |  |  |  | 2 | 0.42 | 0.6595 |
|  |  | Yes | 6155 | 66.20 |  |  |  |
|  |  | No | 1316 | 14.16 |  |  |  |
|  |  | No response | 1826 | 19.64 |  |  |  |
| **10** | **Breed** |  |  |  | 21 | 16.35 | <0.0001 |
|  |  | Romney | 80 | 0.86 |  |  |  |
|  |  | Bentheim | 93 | 1.00 |  |  |  |
|  |  | Charollais | 74 | 0.80 |  |  |  |
|  |  | Dorper | 279 | 3.00 |  |  |  |
|  |  | Coburg | 125 | 1.34 |  |  |  |
|  |  | Ile-De-France | 212 | 2.28 |  |  |  |
|  |  | Leine | 513 | 5.52 |  |  |  |
|  |  | German Mutton Merino | 156 | 1.68 |  |  |  |
|  |  | German Merino-Mix | 730 | 7.85 |  |  |  |
|  |  | German Merino | 1311 | 14.10 |  |  |  |
|  |  | Merino Longwool | 251 | 2.70 |  |  |  |
|  |  | White Polled Heath | 192 | 2.07 |  |  |  |
|  |  | East Friesian | 230 | 2.47 |  |  |  |
|  |  | German Blackheaded Mutton | 681 | 7.32 |  |  |  |
|  |  | Suffolk | 725 | 7.80 |  |  |  |
|  |  | Swifter | 147 | 1.58 |  |  |  |
|  |  | Texel | 679 | 7.30 |  |  |  |
|  |  | German Whiteheaded Mutton | 224 | 2.41 |  |  |  |
|  |  | German Grey Heath | 334 | 3.59 |  |  |  |
|  |  | Pomeranian Coarsewool | 98 | 1.05 |  |  |  |
|  |  | German White Heath | 178 | 1.91 |  |  |  |
|  |  | Others | 1985 | 21.35 |  |  |  |

**Table S5.** Odds ratios (OR) and 95%-confidence intervals (CI) for the footrot scores found on sheep at the time of sampling using an ordered multinomial distribution function with a cumulative logit link function. For each category the reference (Ref) for OR is 1.

| **Source of variation** | **Category** | **OR** | **CI** | |
| --- | --- | --- | --- | --- |
| **Region** |  |  |  |  |
|  | Northern Germany | 1 | Ref | |
|  | Eastern Germany | 0.739 | 0.304 | 1.794 |
|  | Southern Germany | 0.488 | 0.188 | 1.267 |
| **Month of recording** |  |  |  |  |
|  | 1 (Jan-Feb) | 1 | Ref | |
|  | 2 (Mar-Apr) | 7.221 | 3.212 | 16.230 |
|  | 3 (May-Jun) | 3.671 | 1.136 | 11.867 |
|  | 4 (Jul-Aug) | 7.215 | 3.151 | 16.521 |
|  | 5 (Sep-Oct) | 2.631 | 1.405 | 4.925 |
|  | 6 (Nov-Dec) | 3.500 | 1.542 | 7.944 |
| **Year** |  |  |  |  |
|  | 2019 | 1 | Ref |  |
|  | 2020 | 0.181 | 0.105 | 0.313 |
| **Sex of the animal** |  |  |  |  |
|  | Male | 1 | Ref |  |
|  | Female | 1.741 | 1.411 | 2.147 |
| **Age of the animal** |  |  |  |  |
|  | Lamb (<1 year) | 1 | Ref |  |
|  | Sheep (>1 year) | 1.531 | 1.204 | 1.947 |
| **Clinical signs of footrot in the flock within the last 12 months** |  |  |  |  |
|  | Yes | 1 | Ref |  |
|  | No | 0.446 | 0.232 | 0.857 |
|  | No Response | 0.679 | 0.322 | 1.431 |
| **Breed** |  |  |  |  |
|  | Romney | 1 | Ref |  |
|  | Bentheim | 13.152 | 3.499 | 49.433 |
|  | Charollais | 9.165 | 2.269 | 37.016 |
|  | Dorper | 5.812 | 1.422 | 23.763 |
|  | Coburg | 7.372 | 2.373 | 22.902 |
|  | Ile-De-France | 1.803 | 0.616 | 5.279 |
|  | Leine | 7.313 | 2.373 | 22.902 |
|  | German Mutton Merino | 23.496 | 7.101 | 77.749 |
|  | German Merino-Mix | 13.037 | 4.577 | 37.133 |
|  | German Merino | 15.140 | 5.461 | 41.977 |
|  | Merino Longwool | 12.878 | 3.528 | 47.001 |
|  | White Polled Heath | 8.325 | 2.349 | 29.511 |
|  | East Friesian | 5.854 | 1.186 | 28.897 |
|  | German Blackheaded Mutton | 6.022 | 2.148 | 16.884 |
|  | Suffolk | 2.858 | 0.973 | 8.393 |
|  | Swifter | 112.289 | 34.373 | 366.823 |
|  | Texel | 10.633 | 3.654 | 30.946 |
|  | German Whiteheaded Mutton | 8.909 | 2.594 | 30.597 |
|  | German Grey Heath | 4.951 | 1.595 | 15.373 |
|  | Pomeranian Coarsewool | 3.469 | 0.953 | 12.630 |
|  | German White Heath | 14.843 | 4.204 | 52.411 |
|  | Others | 4.537 | 1.725 | 11.931 |

**Table S6.** Odds ratios (OR) with their 95% confidence intervals (95%-CI) and p-values of Swifter compared to all other sheep breeds. OR > 1 indicate higher risk of infection with *D. nodosus* in Swifter compared to all other sheep breeds.

| **Breed of sheep** | **P-value** | | **OR** | **95%-CI** | |
| --- | --- | --- | --- | --- | --- |
| Bentheim | | <0.0001 | 803.479 | 141.312 | >999.999 |
| Charollais | | <0.0001 | 356.942 | 51.377 | >999.999 |
| Dorper | | <0.0001 | 592.135 | 82.051 | >999.999 |
| Coburg | | <0.0001 | 661.633 | 132.388 | >999.999 |
| Ile-de-France | | <0.0001 | 910.303 | 208.118 | >999.999 |
| Leine | | <0.0001 | 604.281 | 121.345 | >999.999 |
| German Mutton Merino | | <0.0001 | >999.999 | 231.460 | >999.999 |
| German Merino-Mix | | <0.0001 | 269.309 | 58.493 | >999.999 |
| German Merino | | <0.0001 | 198.208 | 126.041 | >999.999 |
| Merino Longwool | | <0.0001 | >999.999 | 161.096 | >999.999 |
| White Polled Heath | | <0.0001 | >999.999 | 179.607 | >999.999 |
| East Friesian | | <0.0001 | 894.060 | 83.473 | >999.999 |
| Romney | | <0.0001 | >999.999 | >999.999 | >999.999 |
| German Blackheaded Mutton | | <0.0001 | 661.916 | 152.694 | >999.999 |
| Suffolk | | <0.0001 | 621.903 | 136.079 | >999.999 |
| Texel | | <0.0001 | >999.999 | 270.180 | >999.999 |
| German Whiteheaded Mutton | | <0.0001 | >999.999 | 297.010 | >999.999 |
| Others | | <0.0001 | >999.999 | 247.013 | >999.999 |
| German Grey Heath | | <0.0001 | 477.541 | 86.936 | >999.999 |
| Pomeranian Coarsewool | | <0.0001 | 540.546 | 98.359 | >999.999 |
| German White Heath | | <0.0001 | 185.314 | 31.849 | >999.999 |

**Table S7.** Odds ratios (OR) with their 95% confidence intervals (95%-CI) and p-values of Swifter compared to all other sheep breeds. OR > 1 indicate higher risk of infection with virulent *D. nodosus* in Swifter compared to all other sheep breeds.

| **Breed of sheep** | **P-value** | | **OR** | **95%-CI** | |
| --- | --- | --- | --- | --- | --- |
| Bentheim | | <0.0001 | 267.697 | 49.870 | >999.999 |
| Charollais | | <0.0001 | 228.780 | 36.096 | >999.999 |
| Dorper | | <0.0001 | 524.023 | 69.573 | >999.999 |
| Coburg | | <0.0001 | 462.343 | 105.664 | >999.999 |
| Ile-de-France | | <0.0001 | 751.822 | 196.499 | >999.999 |
| Leine | | <0.0001 | 558.702 | 126.590 | >999.999 |
| German Mutton Merino | | <0.0001 | >999.999 | 209.652 | >999.999 |
| German Merino-Mix | | <0.0001 | 198.147 | 49.076 | 800.033 |
| German Merino | | <0.0001 | 400.861 | 115.830 | >999.999 |
| Merino Longwool | | <0.0001 | >999.999 | 166.405 | >999.999 |
| White Polled Heath | | <0.0001 | >999.999 | 249.903 | >999.999 |
| East Friesian | | <0.0001 | 564.886 | 56.018 | >999.999 |
| Romney | | <0.0001 | >999.999 | 978.667 | >999.999 |
| German Blackheaded Mutton | | <0.0001 | 539.224 | 141.786 | >999.999 |
| Suffolk | | <0.0001 | 415.960 | 102.447 | >999.999 |
| Texel | | <0.0001 | >999.999 | 262.029 | >999.999 |
| German Whiteheaded Mutton | | <0.0001 | >999.999 | 408.852 | >999.999 |
| Others | | <0.0001 | 863.118 | 237.524 | >999.999 |
| German Grey Heath | | <0.0001 | 347.403 | 71.757 | >999.999 |
| Pomeranian Coarsewool | | <0.0001 | 428.434 | 89.230 | >999.999 |
| German White Heath | | <0.0001 | 127.029 | 25.057 | >999.999 |

**Table S8.** Odds ratios (OR) with their 95% confidence intervals (95%-CI) and p-values of German White Heath compared to all other sheep breeds. OR >1 (<1) indicate higher (lower) risk of infection with *D. nodosus* in German White Heath compared to the other sheep breeds.

| **Breed of sheep** | **P-value** | **OR** | **95%-CI** | |
| --- | --- | --- | --- | --- |
| Bentheim | 0.0415 | 4.336 | 1.056 | 17.799 |
| Charollais | 0.4439 | 1.926 | 0.360 | 10.317 |
| Dorper | 0.1913 | 3.195 | 0.559 | 18.251 |
| Coburg | 0.0177 | 3.570 | 1.247 | 10.219 |
| Ile-de-France | 0.0064 | 4.912 | 1.565 | 15.416 |
| Leine | 0.0639 | 3.261 | 0.934 | 11.384 |
| German Mutton Merino | 0.0082 | 7.000 | 1.654 | 29.634 |
| German Merino-Mix | 0.5535 | 1.453 | 0.422 | 5.004 |
| German Merino | 0.0845 | 2.688 | 0.874 | 8.270 |
| Merino Longwool | 0.0464 | 10.011 | 1.037 | 96.619 |
| White Polled Heath | 0.0205 | 7.707 | 1.369 | 43.381 |
| East Friesian | 0.1545 | 4.825 | 0.553 | 42.104 |
| Romney | <0.0001 | 81.959 | 12.124 | 554.064 |
| German Blackheaded Mutton | 0.0263 | 3.572 | 1.162 | 10.979 |
| Suffolk | 0.0434 | 3.356 | 1.036 | 10.867 |
| Swifter | <0.0001 | 0.005 | <0.001 | 0.031 |
| Texel | 0.0014 | 6.489 | 2.068 | 20.364 |
| German Whiteheaded Mutton | 0.0024 | 8.909 | 2.170 | 36.577 |
| Others | 0.0016 | 5.519 | 1.914 | 15.917 |
| German Grey Heath | 0.1302 | 2.577 | 0.756 | 8.781 |
| Pomeranian Coarsewool | 0.1338 | 2.917 | 0.720 | 11.822 |

**Table S9.** Odds ratios (OR) with their 95% confidence intervals (95%-CI) and p-values of German White Heath compared to all other sheep breeds. OR > 1 indicate higher risk of infection with virulent *D. nodosus* in German White Heath compared to most other sheep breeds.

| **Breed of sheep** | **P-value** | **OR** | **95%-CI** | |
| --- | --- | --- | --- | --- |
| Bentheim | 0.3035 | 2.107 | 0.509 | 8.719 |
| Charollais | 0.4845 | 1.801 | 0.346 | 9.374 |
| Dorper | 0.1346 | 4.125 | 0.645 | 26.401 |
| Coburg | 0.0103 | 3.640 | 1.356 | 9.766 |
| Ile-de-France | 0.0012 | 5.919 | 2.015 | 17.380 |
| Leine | 0.0156 | 4.398 | 1.325 | 14.601 |
| German Mutton Merino | 0.0026 | 7.979 | 2.064 | 30.850 |
| German Merino-Mix | 0.4566 | 1.560 | 0.484 | 5.029 |
| German Merino | 0.0337 | 3.156 | 1.093 | 9.112 |
| Merino Longwool | 0.0166 | 10.369 | 1.529 | 70.295 |
| White Polled Heath | 0.0012 | 11.522 | 2.638 | 50.327 |
| East Friesian | 0.1750 | 4.447 | 0.515 | 38.430 |
| Romney | <0.0001 | 45.932 | 9.445 | 223.357 |
| German Blackheaded Mutton | 0.0077 | 4.245 | 1.467 | 12.281 |
| Suffolk | 0.0394 | 3.275 | 1.059 | 10.124 |
| Swifter | <0.0001 | 0.008 | 0.002 | 0.040 |
| Texel | 0.0002 | 8.080 | 2.724 | 23.970 |
| German Whiteheaded Mutton | 0.0002 | 17.696 | 4.006 | 78.166 |
| Others | 0.0002 | 6.795 | 2.499 | 18.474 |
| German Grey Heath | 0.0902 | 2.735 | 0.854 | 8.757 |
| Pomeranian Coarsewool | 0.0725 | 3.373 | 0.895 | 12.712 |

**Table S10.** Odds ratios (OR) with their 95% confidence intervals (95%-CI) and p-values of German Grey Heath compared to all other sheep breeds. OR > 1 indicate higher risk of infection with *D. nodosus* in German Grey Heath compared to some other sheep breeds.

| **Breed of sheep** | **P-value** | **OR** | **95%-CI** | |
| --- | --- | --- | --- | --- |
| Bentheim | 0.4592 | 1.683 | 0.424 | 6.674 |
| Charollais | 0.7266 | 0.747 | 0.146 | 3.823 |
| Dorper | 0.8025 | 1.240 | 0.230 | 6.690 |
| Coburg | 0.5839 | 1.385 | 0.431 | 4.451 |
| Ile-de-France | 0.2336 | 1.906 | 0.659 | 5.511 |
| Leine | 0.7017 | 1.265 | 0.379 | 4.221 |
| German Mutton Merino | 0.1561 | 2.717 | 0.683 | 10.811 |
| German Merino-Mix | 0.3273 | 0.564 | 0.179 | 1.774 |
| German Merino | 0.9357 | 1.043 | 0.373 | 2.922 |
| Merino Longwool | 0.2318 | 3.885 | 0.420 | 35.926 |
| White Polled Heath | 0.1075 | 2.991 | 0.788 | 11.355 |
| East Friesian | 0.5620 | 1.872 | 0.225 | 15.599 |
| Romney | 0.0003 | 31.805 | 4.946 | 204.506 |
| German Blackheaded Mutton | 0.5394 | 1.386 | 0.489 | 3.932 |
| Suffolk | 0.6386 | 1.302 | 0.432 | 3.922 |
| Swifter | <0.0001 | 0.002 | <0.001 | 0.012 |
| Texel | 0.0896 | 2.518 | 0.867 | 7.316 |
| German Whiteheaded Mutton | 0.0721 | 3.457 | 0.895 | 13.359 |
| Others | 0.1241 | 2.142 | 0.811 | 5.655 |
| Pomeranian Coarsewool | 0.8563 | 1.132 | 0.296 | 4.329 |
| German White Heath | 0.1302 | 0.388 | 0.114 | 1.322 |

**Table S11.** Odds ratios (OR) with their 95% confidence intervals (95%-CI) and p-values of German Grey Heath compared to all other sheep breeds. OR > 1 indicate higher risk of infection with virulent *D. nodosus* in German Grey Heath compared to some other sheep breeds.

| **Breed of sheep** | **P-value** | **OR** | **95%-CI** | |
| --- | --- | --- | --- | --- |
| Bentheim | 0.6498 | 0.712 | 0.164 | 3.087 |
| Charollais | 0.6056 | 0.641 | 0.119 | 3.465 |
| Dorper | 0.6935 | 1.462 | 0.221 | 9.659 |
| Coburg | 0.6957 | 1.265 | 0.389 | 4.117 |
| Ile-de-France | 0.1956 | 2.031 | 0.694 | 5.943 |
| Leine | 0.5322 | 1.482 | 0.431 | 5.094 |
| German Mutton Merino | 0.1445 | 2.818 | 0.701 | 11.331 |
| German Merino-Mix | 0.2785 | 0.524 | 0.163 | 1.686 |
| German Merino | 0.8594 | 1.099 | 0.388 | 3.114 |
| Merino Longwool | 0.2073 | 3.660 | 0.487 | 27.503 |
| White Polled Heath | 0.0274 | 4.087 | 1.170 | 14.277 |
| East Friesian | 0.6014 | 1.773 | 0.207 | 15.214 |
| Romney | 0.0013 | 14.194 | 2.813 | 71.634 |
| German Blackheaded Mutton | 0.4660 | 1.481 | 0.515 | 4.260 |
| Suffolk | 0.8597 | 1.107 | 0.357 | 3.433 |
| Swifter | <0.0001 | 0.003 | <0.001 | 0.016 |
| Texel | 0.0629 | 2.799 | 0.946 | 8.282 |
| German Whiteheaded Mutton | 0.0258 | 5.454 | 1.228 | 24.224 |
| Others | 0.0957 | 2.305 | 0.863 | 6.156 |
| Pomeranian Coarsewool | 0.9472 | 1.047 | 0.270 | 4.056 |
| German White Heath | 0.1039 | 0.357 | 0.103 | 1.235 |

**Table S12.** Odds ratios (OR) with their 95% confidence intervals (95%-CI) and p-values of White Polled Heath compared to all other sheep breeds. OR < 1 indicate lower risk of infection with *D. nodosus* in White Polled Heath compared to some other sheep breeds.

| **Breed of sheep** | **P-value** | **OR** | **95%-CI** | |
| --- | --- | --- | --- | --- |
| Bentheim | 0.5328 | 0.563 | 0.092 | 3.430 |
| Charollais | 0.1761 | 0.250 | 0.034 | 1.863 |
| Dorper | 0.3999 | 0.415 | 0.053 | 3.221 |
| Coburg | 0.3643 | 0.463 | 0.088 | 2.442 |
| Ile-de-France | 0.5780 | 0.637 | 0.130 | 3.116 |
| Leine | 0.3163 | 0.423 | 0.079 | 2.276 |
| German Mutton Merino | 0.9171 | 0.908 | 0.148 | 5.562 |
| German Merino-Mix | 0.0476 | 0.189 | 0.036 | 0.982 |
| German Merino | 0.1883 | 0.349 | 0.073 | 1.675 |
| Merino Longwool | 0.8386 | 1.299 | 0.105 | 16.082 |
| East Friesian | 0.7048 | 0.626 | 0.055 | 7.066 |
| Romney | 0.0351 | 10.634 | 1.179 | 95.888 |
| German Blackheaded Mutton | 0.3374 | 0.463 | 0.096 | 2.231 |
| Suffolk | 0.3106 | 0.435 | 0.087 | 2.173 |
| Swifter | <0.0001 | <0.001 | <0.001 | 0.006 |
| Texel | 0.8314 | 0.842 | 0.173 | 4.102 |
| German Whiteheaded Mutton | 0.8735 | 1.156 | 0.194 | 6.886 |
| Others | 0.6684 | 0.716 | 0.155 | 3.301 |
| German Grey Heath | 0.1075 | 0.334 | 0.088 | 1.269 |
| Pomeranian Coarsewool | 0.2854 | 0.378 | 0.064 | 2.250 |
| German White Heath | 0.0205 | 0.130 | 0.023 | 0.730 |

**Table S13.** Odds ratios (OR) with their 95% confidence intervals (95%-CI) and p-values of White Polled Heath compared to all other sheep breeds. OR < 1 indicate lower risk of infection with virulent *D. nodosus* in White Polled Heath compared to some other sheep breeds.

| **Breed of sheep** | **P-value** | **OR** | **95%-CI** | |
| --- | --- | --- | --- | --- |
| Bentheim | 0.0308 | 0.183 | 0.039 | 0.855 |
| Charollais | 0.0427 | 0.156 | 0.026 | 0.941 |
| Dorper | 0.3083 | 0.358 | 0.050 | 2.583 |
| Coburg | 0.1014 | 0.316 | 0.080 | 1.254 |
| Ile-de-France | 0.3099 | 0.514 | 0.142 | 1.858 |
| Leine | 0.1821 | 0.382 | 0.093 | 1.571 |
| German Mutton Merino | 0.6368 | 0.693 | 0.151 | 3.183 |
| German Merino-Mix | 0.0040 | 0.135 | 0.035 | 0.529 |
| German Merino | 0.0457 | 0.274 | 0.077 | 0.976 |
| Merino Longwool | 0.9192 | 0.900 | 0.117 | 6.904 |
| East Friesian | 0.4102 | 0.386 | 0.040 | 3.720 |
| Romney | 0.1169 | 3.987 | 0.708 | 22.459 |
| German Blackheaded Mutton | 0.1252 | 0.368 | 0.103 | 1.320 |
| Suffolk | 0.0642 | 0.284 | 0.075 | 1.077 |
| Swifter | <0.0001 | <0.001 | <0.001 | 0.004 |
| Texel | 0.5915 | 0.701 | 0.192 | 2.563 |
| German Whiteheaded Mutton | 0.6077 | 1.536 | 0.298 | 7.907 |
| Others | 0.367 | 0.590 | 0.174 | 2.000 |
| German Grey Heath | 0.0161 | 0.237 | 0.074 | 0.766 |
| Pomeranian Coarsewool | 0.1097 | 0.293 | 0.065 | 1.319 |
| German White Heath | 0.0012 | 0.087 | 0.020 | 0.379 |

**Table S14.** Odds ratios (OR) with their 95% confidence intervals (95%-CI) and p-values of Merino Longwool compared to all other sheep breeds. OR < 1 indicate lower risk of infection with *D. nodosus* in Merino Longwool compared to some other sheep breeds.

| **Breed of sheep** | **P-value** | **OR** | **95%-CI** | |
| --- | --- | --- | --- | --- |
| Bentheim | 0.4669 | 0.433 | 0.045 | 4.128 |
| Charollais | 0.1795 | 0.192 | 0.017 | 2.135 |
| Dorper | 0.3558 | 0.319 | 0.028 | 3.604 |
| Coburg | 0.3474 | 0.357 | 0.042 | 3.065 |
| Ile-de-France | 0.4970 | 0.491 | 0.063 | 3.829 |
| Leine | 0.3052 | 0.326 | 0.038 | 2.780 |
| German Mutton Merino | 0.7544 | 0.699 | 0.074 | 6.575 |
| German Merino-Mix | 0.0713 | 0.145 | 0.018 | 1.182 |
| German Merino | 0.2048 | 0.269 | 0.035 | 2.050 |
| White Polled Heath | 0.8386 | 0.770 | 0.062 | 9.533 |
| East Friesian | 0.6060 | 0.482 | 0.030 | 7.723 |
| Romney | 0.1105 | 8.187 | 0.619 | 108.316 |
| German Blackheaded Mutton | 0.3248 | 0.357 | 0.046 | 2.776 |
| Suffolk | 0.3041 | 0.335 | 0.042 | 2.696 |
| Swifter | <0.0001 | <0.001 | <0.001 | 0.006 |
| Texel | 0.6812 | 0.648 | 0.082 | 5.129 |
| German Whiteheaded Mutton | 0.9184 | 0.890 | 0.096 | 8.289 |
| Others | 0.5618 | 0.551 | 0.074 | 4.121 |
| German Grey Heath | 0.2318 | 0.257 | 0.028 | 2.381 |
| Pomeranian Coarsewool | 0.2766 | 0.291 | 0.032 | 2.686 |
| German White Heath | 0.0464 | 0.100 | 0.010 | 0.964 |

**Table S15.** Odds ratios (OR) with their 95% confidence intervals (95%-CI) and p-values of Merino Longwool compared to all other sheep breeds. OR < 1 indicate lower risk of infection with virulent *D. nodosus* in Merino Longwool compared to some other sheep breeds.

| **Breed of sheep** | **P-value** | **OR** | **95%-CI** | |
| --- | --- | --- | --- | --- |
| Bentheim | 0.1123 | 0.203 | 0.028 | 1.453 |
| Charollais | 0.1027 | 0.174 | 0.021 | 1.422 |
| Dorper | 0.4225 | 0.398 | 0.042 | 3.784 |
| Coburg | 0.2521 | 0.351 | 0.059 | 2.106 |
| Ile-de-France | 0.5143 | 0.571 | 0.106 | 3.079 |
| Leine | 0.3493 | 0.424 | 0.070 | 2.556 |
| German Mutton Merino | 0.7836 | 0.770 | 0.119 | 4.994 |
| German Merino-Mix | 0.0328 | 0.150 | 0.026 | 0.857 |
| German Merino | 0.1606 | 0.304 | 0.058 | 1.604 |
| White Polled Heath | 0.9192 | 1.111 | 0.145 | 5.525 |
| East Friesian | 0.5122 | 0.429 | 0.034 | 5.394 |
| Romney | 0.1555 | 4.430 | 0.568 | 34.547 |
| German Blackheaded Mutton | 0.2979 | 0.409 | 0.076 | 2.200 |
| Suffolk | 0.1894 | 0.316 | 0.056 | 1.766 |
| Swifter | <0.0001 | <0.001 | <0.001 | 0.006 |
| Texel | 0.7736 | 0.779 | 0.142 | 4.263 |
| German Whiteheaded Mutton | 0.5970 | 1.707 | 0.235 | 12.383 |
| Others | 0.6134 | 0.655 | 0.127 | 3.377 |
| German Grey Heath | 0.1644 | 0.264 | 0.040 | 1.726 |
| Pomeranian Coarsewool | 0.2391 | 0.325 | 0.050 | 2.110 |
| German White Heath | 0.0166 | 0.096 | 0.014 | 0.654 |

**Table S16.** Odds ratios (OR) with their 95% confidence intervals (95%-CI) and p-values of German Mutton Merino compared to all other sheep breeds. OR < 1 indicate lower risk of infection with *D. nodosus* in German Mutton Merino compared to some other sheep breeds.

| **Breed of sheep** | **P-value** | **OR** | **95%-CI** | |
| --- | --- | --- | --- | --- |
| Bentheim | 0.5068 | 0.619 | 0.151 | 2.548 |
| Charollais | 0.1239 | 0.275 | 0.053 | 1.424 |
| Dorper | 0.3677 | 0.456 | 0.083 | 2.515 |
| Coburg | 0.2905 | 0.510 | 0.146 | 1.777 |
| Ile-de-France | 0.5172 | 0.702 | 0.240 | 2.050 |
| Leine | 0.2192 | 0.466 | 0.138 | 1.576 |
| German Merino-Mix | 0.0098 | 0.208 | 0.063 | 0.684 |
| German Merino | 0.0791 | 0.384 | 0.132 | 1.118 |
| Merino Longwool | 0.7544 | 1.430 | 0.152 | 13.446 |
| White Polled Heath | 0.9171 | 1.101 | 0.180 | 6.742 |
| East Friesian | 0.7339 | 0.689 | 0.081 | 5.895 |
| Romney | 0.0107 | 11.708 | 1.769 | 77.489 |
| German Blackheaded Mutton | 0.1510 | 0.510 | 0.204 | 1.278 |
| Suffolk | 0.1959 | 0.479 | 0.157 | 1.461 |
| Swifter | <0.0001 | <0.001 | <0.001 | 0.004 |
| Texel | 0.8872 | 0.927 | 0.325 | 2.644 |
| German Whiteheaded Mutton | 0.7300 | 1.273 | 0.324 | 5.005 |
| Others | 0.6450 | 0.788 | 0.287 | 2.168 |
| German Grey Heath | 0.1561 | 0.368 | 0.093 | 1.465 |
| Pomeranian Coarsewool | 0.2118 | 0.417 | 0.105 | 1.647 |
| German White Heath | 0.0082 | 0.143 | 0.034 | 0.605 |

**Table S17.** Odds ratios (OR) with their 95% confidence intervals (95%-CI) and p-values of German Mutton Merino compared to all other sheep breeds. OR < 1 indicate lower risk of infection with virulent *D. nodosus* in German Mutton Merino compared to some other sheep breeds.

| **Breed of sheep** | **P-value** | **OR** | **95%-CI** | |
| --- | --- | --- | --- | --- |
| Bentheim | 0.0665 | 0.264 | 0.064 | 1.095 |
| Charollais | 0.0687 | 0.226 | 0.045 | 1.121 |
| Dorper | 0.4758 | 0.517 | 0.084 | 3.170 |
| Coburg | 0.1874 | 0.456 | 0.142 | 1.465 |
| Ile-de-France | 0.5545 | 0.742 | 0.275 | 1.998 |
| Leine | 0.3103 | 0.551 | 0.174 | 1.742 |
| German Merino-Mix | 0.0041 | 0.195 | 0.064 | 0.595 |
| German Merino | 0.0661 | 0.395 | 0.147 | 1.064 |
| Merino Longwool | 0.7836 | 1.299 | 0.200 | 8.433 |
| White Polled Heath | 0.6368 | 1.444 | 0.314 | 6.637 |
| East Friesian | 0.5906 | 0.557 | 0.066 | 4.691 |
| Romney | 0.0263 | 5.756 | 1.229 | 26.968 |
| German Blackheaded Mutton | 0.1440 | 0.532 | 0.228 | 1.241 |
| Suffolk | 0.0959 | 0.410 | 0.144 | 1.171 |
| Swifter | <0.0001 | <0.001 | <0.001 | 0.005 |
| Texel | 0.9798 | 1.013 | 0.383 | 2.678 |
| German Whiteheaded Mutton | 0.2749 | 2.218 | 0.531 | 9.266 |
| Others | 0.7364 | 0.852 | 0.334 | 2.171 |
| German Grey Heath | 0.1080 | 0.343 | 0.093 | 1.265 |
| Pomeranian Coarsewool | 0.1900 | 0.423 | 0.117 | 1.532 |
| German White Heath | 0.0026 | 0.125 | 0.032 | 0.485 |

**Table S18.** Odds ratios (OR) with their 95% confidence intervals (95%-CI) and p-values of German Merino compared to all other sheep breeds. OR > 1 indicate higher risk of infection with *D. nodosus* in German Merino compared to some other sheep breeds.

| **Breed of sheep** | **P-value** | **OR** | **95%-CI** | |
| --- | --- | --- | --- | --- |
| Bentheim | 0.3905 | 1.613 | 0.542 | 4.801 |
| Charollais | 0.6382 | 0.716 | 0.178 | 2.876 |
| Dorper | 0.8142 | 1.189 | 0.282 | 5.018 |
| Coburg | 0.5210 | 1.328 | 0.558 | 3.159 |
| Ile-de-France | 0.0400 | 1.827 | 1.028 | 3.248 |
| Leine | 0.6607 | 1.213 | 0.512 | 2.872 |
| German Mutton Merino | 0.0791 | 2.604 | 0.895 | 7.577 |
| German Merino-Mix | 0.0797 | 0.541 | 0.272 | 1.075 |
| Merino Longwool | 0.2048 | 3.724 | 0.488 | 28.418 |
| White Polled Heath | 0.1883 | 2.867 | 0.597 | 13.765 |
| East Friesian | 0.5562 | 1.795 | 0.256 | 12.582 |
| Romney | <0.0001 | 30.486 | 5.796 | 160.336 |
| German Blackheaded Mutton | 0.3209 | 1.329 | 0.758 | 2.328 |
| Suffolk | 0.5325 | 1.248 | 0.622 | 2.505 |
| Swifter | <0.0001 | 0.002 | <0.001 | 0.008 |
| Texel | 0.0063 | 2.414 | 1.283 | 4.542 |
| German Whiteheaded Mutton | 0.0262 | 3.314 | 1.153 | 9.526 |
| Others | 0.0007 | 2.053 | 1.354 | 3.112 |
| German Grey Heath | 0.9357 | 0.959 | 0.342 | 2.684 |
| Pomeranian Coarsewool | 0.8773 | 1.085 | 0.385 | 3.055 |
| German White Heath | 0.0845 | 0.372 | 0.121 | 1.144 |

**Table S19.** Odds ratios (OR) with their 95% confidence intervals (95%-CI) and p-values of German Merino compared to all other sheep breeds. OR > 1 indicate higher risk of infection with virulent *D. nodosus* in German Merino compared to some other sheep breeds.

| **Breed of sheep** | **P-value** | **OR** | **95%-CI** | |
| --- | --- | --- | --- | --- |
| Bentheim | 0.4872 | 0.648 | 0.190 | 2.204 |
| Charollais | 0.4653 | 0.584 | 0.137 | 2.477 |
| Dorper | 0.7368 | 1.330 | 0.252 | 7.035 |
| Coburg | 0.7517 | 1.152 | 0.480 | 2.763 |
| Ile-de-France | 0.0376 | 1.849 | 1.036 | 3.300 |
| Leine | 0.5098 | 1.349 | 0.554 | 3.284 |
| German Mutton Merino | 0.0844 | 2.565 | 0.880 | 7.475 |
| German Merino-Mix | 0.0405 | 0.477 | 0.235 | 0.969 |
| Merino Longwool | 0.1892 | 3.331 | 0.553 | 20.081 |
| White Polled Heath | 0.0559 | 3.720 | 0.967 | 14.305 |
| East Friesian | 0.6346 | 1.614 | 0.224 | 11.621 |
| Romney | 0.0002 | 12.919 | 3.295 | 50.659 |
| German Blackheaded Mutton | 0.3006 | 1.348 | 0.766 | 2.373 |
| Suffolk | 0.9830 | 1.008 | 0.488 | 2.082 |
| Swifter | <0.0001 | 0.003 | <0.001 | 0.010 |
| Texel | 0.0044 | 2.548 | 1.338 | 4.849 |
| German Whiteheaded Mutton | 0.0101 | 4.964 | 1.463 | 16.835 |
| Others | 0.0005 | 2.098 | 1.380 | 3.190 |
| German Grey Heath | 0.8594 | 0.910 | 0.321 | 2.580 |
| Pomeranian Coarsewool | 0.9273 | 0.953 | 0.336 | 2.699 |
| German White Heath | 0.0521 | 0.325 | 0.104 | 1.010 |

**Table S20.** Odds ratios (OR) with their 95% confidence intervals (95%-CI) and p-values of German Blackheaded Mutton compared to all other sheep breeds. OR > 1 indicate higher risk of infection with *D. nodosus* in German Blackheaded Mutton compared to some other sheep breeds.

| **Breed of sheep** | **P-value** | **OR** | **95%-CI** | |
| --- | --- | --- | --- | --- |
| Bentheim | 0.7268 | 1.214 | 0.409 | 3.601 |
| Charollais | 0.3778 | 0.539 | 0.137 | 2.127 |
| Dorper | 0.8802 | 0.895 | 0.210 | 3.808 |
| Coburg | 0.9992 | 1.000 | 0.423 | 2.360 |
| Ile-de-France | 0.2703 | 1.375 | 0.780 | 2.424 |
| Leine | 0.8263 | 0.913 | 0.405 | 2.060 |
| German Mutton Merino | 0.1510 | 1.960 | 0.782 | 4.911 |
| German Merino-Mix | 0.0223 | 0.407 | 0.188 | 0.880 |
| German Merino | 0.3209 | 0.753 | 0.429 | 1.319 |
| Merino Longwool | 0.3248 | 2.803 | 0.360 | 21.804 |
| White Polled Heath | 0.3374 | 2.158 | 0.448 | 10.387 |
| East Friesian | 0.7621 | 1.351 | 0.193 | 9.460 |
| Romney | 0.0002 | 22.946 | 4.362 | 120.699 |
| Suffolk | 0.8504 | 0.940 | 0.491 | 1.797 |
| Swifter | <0.0001 | 0.002 | <0.001 | 0.007 |
| Texel | 0.0244 | 1.817 | 1.080 | 3.055 |
| German Whiteheaded Mutton | 0.0813 | 2.494 | 0.892 | 6.971 |
| Others | 0.0545 | 1.545 | 0.992 | 2.408 |
| German Grey Heath | 0.5394 | 0.721 | 0.254 | 2.047 |
| Pomeranian Coarsewool | 0.7008 | 0.817 | 0.291 | 2.295 |
| German White Heath | 0.0263 | 0.280 | 0.091 | 0.861 |

**Table S21.** Odds ratios (OR) with their 95% confidence intervals (95%-CI) and p-values of German Blackheaded Mutton compared to all other sheep breeds. OR > 1 indicate higher risk of infection with virulent *D. nodosus* in German Blackheaded Mutton compared to some other sheep breeds.

| **Breed of sheep** | **P-value** | **OR** | **95%-CI** | |
| --- | --- | --- | --- | --- |
| Bentheim | 0.2331 | 0.4508 | 0.496 | 1.570 |
| Charollais | 0.2195 | 0.424 | 0.108 | 1.667 |
| Dorper | 0.9723 | 0.972 | 0.194 | 4.871 |
| Coburg | 0.7111 | 0.857 | 0.380 | 1.935 |
| Ile-de-France | 0.2156 | 1.394 | 0.824 | 2.360 |
| Leine | 0.9297 | 1.036 | 0.471 | 2.280 |
| German Mutton Merino | 0.1440 | 1.880 | 0.806 | 4.384 |
| German Merino-Mix | 0.0073 | 0.367 | 0.177 | 0.763 |
| German Merino | 0.2686 | 0.743 | 0.440 | 1.257 |
| Merino Longwool | 0.2979 | 2.443 | 0.455 | 13.127 |
| White Polled Heath | 0.1252 | 2.714 | 0.757 | 9.726 |
| East Friesian | 0.9629 | 1.048 | 0.148 | 7.432 |
| Romney | 0.0003 | 10.820 | 2.953 | 39.650 |
| Suffolk | 0.4210 | 0.771 | 0.410 | 1.452 |
| Swifter | <0.0001 | 0.002 | <0.001 | 0.007 |
| Texel | 0.0100 | 1.904 | 1.166 | 3.107 |
| German Whiteheaded Mutton | 0.0159 | 4.169 | 1.306 | 13.307 |
| Others | 0.0266 | 1.601 | 1.056 | 2.426 |
| German Grey Heath | 0.3901 | 0.644 | 0.236 | 1.756 |
| Pomeranian Coarsewool | 0.6452 | 0.795 | 0.298 | 2.115 |
| German White Heath | 0.0077 | 0.236 | 0.081 | 0.682 |

**Table S22.** Odds ratios (OR) with their 95% confidence intervals (95%-CI) and p-values of Suffolk compared to all other sheep breeds. OR > 1 indicate higher risk of infection with *D. nodosus* in Suffolk compared to some other sheep breeds.

| **Breed of sheep** | **P-value** | **OR** | **95%-CI** | |
| --- | --- | --- | --- | --- |
| Bentheim | 0.6571 | 1.292 | 0.417 | 4.004 |
| Charollais | 0.4226 | 0.574 | 0.148 | 2.230 |
| Dorper | 0.9489 | 0.952 | 0.213 | 4.264 |
| Coburg | 0.8955 | 1.064 | 0.422 | 2.682 |
| Ile-de-France | 0.2993 | 1.464 | 0.713 | 3.005 |
| Leine | 0.9499 | 0.972 | 0.396 | 2.383 |
| German Mutton Merino | 0.1959 | 2.086 | 0.685 | 6.357 |
| German Merino-Mix | 0.0585 | 0.433 | 0.182 | 1.031 |
| German Merino | 0.5325 | 0.801 | 0.399 | 1.608 |
| Merino Longwool | 0.3041 | 2.983 | 0.371 | 23.986 |
| White Polled Heath | 0.3106 | 2.297 | 0.460 | 11.459 |
| East Friesian | 0.7198 | 1.438 | 0.198 | 10.452 |
| Romney | 0.0002 | 24.422 | 4.443 | 134.249 |
| German Blackheaded Mutton | 0.8504 | 1.064 | 0.557 | 2.035 |
| Swifter | <0.0001 | 0.002 | <0.001 | 0.007 |
| Texel | 0.0236 | 1.934 | 1.093 | 3.422 |
| German Whiteheaded Mutton | 0.0647 | 2.655 | 0.942 | 7.480 |
| Others | 0.0987 | 1.645 | 0.911 | 2.969 |
| German Grey Heath | 0.6386 | 0.768 | 0.255 | 2.312 |
| Pomeranian Coarsewool | 0.8006 | 0.869 | 0.293 | 2.580 |
| German White Heath | 0.0434 | 0.298 | 0.092 | 0.965 |

**Table S23.** Odds ratios (OR) with their 95% confidence intervals (95%-CI) and p-values of Suffolk compared to all other sheep breeds. OR > 1 indicate higher risk of infection with virulent *D. nodosus* in Suffolk compared to some other sheep breeds.

| **Breed of sheep** | **P-value** | **OR** | **95%-CI** | |
| --- | --- | --- | --- | --- |
| Bentheim | 0.4770 | 0.644 | 0.191 | 2.168 |
| Charollais | 0.3776 | 0.550 | 0.146 | 2.076 |
| Dorper | 0.7850 | 1.260 | 0.240 | 6.622 |
| Coburg | 0.8178 | 1.112 | 0.452 | 2.732 |
| Ile-de-France | 0.965 | 1.807 | 0.899 | 3.632 |
| Leine | 0.5170 | 1.343 | 0.550 | 3.279 |
| German Mutton Merino | 0.0959 | 2.437 | 0.854 | 6.952 |
| German Merino-Mix | 0.0847 | 0.476 | 0.205 | 1.107 |
| German Merino | 0.9152 | 0.964 | 0.488 | 1.904 |
| Merino Longwool | 0.1894 | 3.167 | 0.566 | 17.707 |
| White Polled Heath | 0.0642 | 3.519 | 0.928 | 13.337 |
| East Friesian | 0.7644 | 1.358 | 0.183 | 10.051 |
| Romney | 0.0001 | 14.027 | 3.591 | 54.796 |
| German Blackheaded Mutton | 0.4210 | 1.296 | 0.689 | 2.439 |
| Swifter | <0.0001 | 0.002 | <0.001 | 0.010 |
| Texel | 0.0014 | 2.468 | 1.418 | 4.294 |
| German Whiteheaded Mutton | 0.0049 | 5.404 | 1.668 | 17.513 |
| Others | 0.0152 | 2.075 | 1.151 | 3.741 |
| German Grey Heath | 0.7429 | 0.835 | 0.285 | 2.450 |
| Pomeranian Coarsewool | 0.9560 | 1.030 | 0.361 | 2.941 |
| German White Heath | 0.0394 | 0.305 | 0.099 | 0.944 |

**Table S24.** Odds ratios (OR) with their 95% confidence intervals (95%-CI) and p-values of Texel sheep compared to all other sheep breeds. OR < 1 indicate lower risk of infection with *D. nodosus* in Texel sheep compared to some other sheep breeds.

| **Breed of sheep** | **P-value** | **OR** | **95%-CI** | |
| --- | --- | --- | --- | --- |
| Bentheim | 0.4729 | 0.668 | 0.222 | 2.010 |
| Charollais | 0.0817 | 0.297 | 0.076 | 1.165 |
| Dorper | 0.3447 | 0.492 | 0.113 | 2.141 |
| Coburg | 0.1868 | 0.550 | 0.227 | 1.336 |
| Ile-de-France | 0.4013 | 0.757 | 0.395 | 1.450 |
| Leine | 0.1182 | 0.503 | 0.212 | 1.192 |
| German Mutton Merino | 0.8872 | 1.079 | 0.378 | 3.077 |
| German Merino-Mix | 0.0003 | 0.224 | 0.099 | 0.507 |
| German Merino | 0.0063 | 0.414 | 0.220 | 0.780 |
| Merino Longwool | 0.6812 | 1.543 | 0.195 | 12.206 |
| White Polled Heath | 0.8314 | 1.188 | 0.244 | 5.787 |
| East Friesian | 0.7669 | 0.743 | 0.105 | 5.280 |
| Romney | 0.0031 | 12.630 | 2.357 | 67.668 |
| German Blackheaded Mutton | 0.0244 | 0.550 | 0.327 | 0.926 |
| Suffolk | 0.0236 | 0.517 | 0.292 | 0.915 |
| Swifter | <0.0001 | <0.001 | <0.001 | 0.004 |
| German Whiteheaded Mutton | 0.5346 | 1.373 | 0.505 | 3.733 |
| Others | 0.5353 | 0.851 | 0.510 | 1.419 |
| German Grey Heath | 0.0896 | 0.397 | 0.137 | 1.154 |
| Pomeranian Coarsewool | 0.1365 | 0.450 | 0.157 | 1.288 |
| German White Heath | 0.0014 | 0.154 | 0.049 | 0.484 |

**Table S25.** Odds ratios (OR) with their 95% confidence intervals (95%-CI) and p-values of Texel sheep compared to all other sheep breeds. OR < 1 indicate lower risk of infection with virulent *D. nodosus* in Texel sheep compared to some other sheep breeds.

| **Breed of sheep** | **P-value** | **OR** | **95%-CI** | |
| --- | --- | --- | --- | --- |
| Bentheim | 0.0248 | 0.261 | 0.081 | 0.844 |
| Charollais | 0.0299 | 0.223 | 0.058 | 0.864 |
| Dorper | 0.4189 | 0.511 | 0.100 | 2.607 |
| Coburg | 0.0655 | 0.450 | 0.193 | 1.052 |
| Ile-de-France | 0.3191 | 0.732 | 0.397 | 1.352 |
| Leine | 0.1581 | 0.544 | 0.234 | 1.267 |
| German Mutton Merino | 0.9798 | 0.987 | 0.373 | 2.612 |
| German Merino-Mix | <0.0001 | 0.193 | 0.088 | 0.422 |
| German Merino | 0.0021 | 0.391 | 0.214 | 0.712 |
| Merino Longwool | 0.7736 | 1.283 | 0.235 | 7.018 |
| White Polled Heath | 0.5915 | 1.426 | 0.390 | 5.211 |
| East Friesian | 0.5533 | 0.550 | 0.076 | 3.964 |
| Romney | 0.0101 | 5.684 | 1.513 | 21.355 |
| German Blackheaded Mutton | 0.0100 | 0.525 | 0.322 | 0.857 |
| Suffolk | 0.0014 | 0.405 | 0.233 | 0.705 |
| Swifter | <0.0001 | <0.001 | <0.001 | 0.004 |
| German Whiteheaded Mutton | 0.1781 | 2.190 | 0.700 | 6.856 |
| Others | 0.4892 | 0.841 | 0.515 | 1.374 |
| German Grey Heath | 0.0394 | 0.338 | 0.121 | 0.949 |
| Pomeranian Coarsewool | 0.0881 | 0.417 | 0.153 | 1.139 |
| German White Heath | 0.0002 | 0.124 | 0.042 | 0.367 |

**Table S26.** On-farm questionnaire for recording of management data.

**On-Farm Questionnaire**

**General Information**

Date: _____________________________

Surname, Name: ______________________________________

Address: _____________________________________________

Postal code, City: ______________________________________

Telephone number: _______________________ Mobil number: ___________________________

E-Mail address: _______________________________________

**Farm specific data:**

1. Are you a member of a herdbook breeding organization? □ Yes □ No
2. Flock size: Number of ewes/rams/lambs: __________ / __________ / __________
3. Sheep breed or mixes of breeds:

Breed 1: _______________________

Breed 2: _______________________

Breed 3: _______________________

Mixes: _______________________________________________________________

1. Further animal species present on farm:

- Goat □ Yes □ No
- Cattle □ Yes □ No
- Horse □ Yes □ No
- Donkey □ Yes □ No

1. Within the last 12 months:

- Have you observed clinical signs of footrot on animals in the flock? □ Yes □ No
- Have you treated diseased sheep with antibiotics for footrot? □ Yes □ No
- Have you treated sheep with footbaths? □ Yes □ No
- Have you treated sheep with a vaccination against footrot? □ Yes □ No

1. Within the last 3-10 years:

- Have you observed clinical signs of footrot on animals in the flock? □ Yes □ No
- Have you treated diseased sheep with antibiotics for footrot? □ Yes □ No
- Have you treated sheep with footbaths? □ Yes □ No
- Have you treated sheep with a vaccination against footrot? □ Yes □ No

**Table S27.** Distribution of breeds per farm. Number of farms that keep one, two, three or more than three sheep breeds.

| **Breeds per farm** | **No. of farms** |
| --- | --- |
| 1 | 136 |
| 2 | 39 |
| 3 | 21 |
| > 3 | 12 |
| Total | 208 |

**Table S28.** Number of flocks in which the respective breed was present at the time of sampling and distribution of sampled animals across the different breeds.

| **Breed** | **No. of flocks** | **No. of sheep** |
| --- | --- | --- |
| Romney | 3 | 80 |
| Bentheim | 7 | 93 |
| Charollais | 12 | 74 |
| Dorper | 4 | 279 |
| Coburg | 16 | 125 |
| Ile-De-France | 5 | 212 |
| Leine | 11 | 513 |
| German Mutton Merino | 4 | 156 |
| German Merino-Mix | 11 | 730 |
| German Merino | 19 | 1311 |
| Merino Longwool | 3 | 251 |
| White Polled Heath | 5 | 192 |
| East Friesian | 4 | 230 |
| German Blackheaded Mutton | 28 | 681 |
| Suffolk | 45 | 725 |
| Swifter | 2 | 147 |
| Texel | 41 | 679 |
| German Whiteheaded Mutton | 15 | 224 |
| German Grey Heath | 8 | 334 |
| Pomeranian Coarsewool | 10 | 98 |
| German White Heath | 5 | 178 |
| Others | 76 | 1985 |
